# Effect of adipokine and ghrelin levels on BMD and fracture risk: an updated systematic review and meta-analysis

**DOI:** 10.3389/fendo.2023.1044039

**Published:** 2023-04-26

**Authors:** Seoyul Lee, Jeong Hun Kim, Yun Kyung Jeon, Jung Sub Lee, Keunyoung Kim, Sun-Kyung Hwang, Jae Ho Kim, Tae Sik Goh, Yun Hak Kim

**Affiliations:** ^1^ Department of Physiology, School of Medicine, Pusan National University, Yangsan, Republic of Korea; ^2^ Biomedical Research Institute, Pusan National University Hospital, Busan, Republic of Korea; ^3^ College of Nursing, Pusan National University, Yangsan, Republic of Korea; ^4^ Department of Internal Medicine, School of Medicine, Pusan National University, Yangsan, Republic of Korea; ^5^ Department of Orthopaedic Surgery, School of Medicine, Pusan National University, Yangsan, Republic of Korea; ^6^ Department of Nuclear Medicine, School of Medicine, Pusan National University, Yangsan, Republic of Korea; ^7^ Research Institute for Convergence of Biomedical Science and Technology, Pusan National University Yangsan Hospital, Yangsan, Republic of Korea; ^8^ Department of Anatomy, School of Medicine, Pusan National University, Yangsan, Republic of Korea; ^9^ Department of Biomedical Informatics, School of Medicine, Pusan National University, Yangsan, Republic of Korea

**Keywords:** adipokines, ghrelin, bone mineral density, fracture risk, meta-analysis

## Abstract

**Context:**

Circulating adipokines and ghrelin affect bone remodeling by regulating the activation and differentiation of osteoblasts and osteoclasts. Although the correlation between adipokines, ghrelin, and bone mineral density (BMD) has been studied over the decades, its correlations are still controversial. Accordingly, an updated meta-analysis with new findings is needed.

**Objective:**

This study aimed to explore the impact of serum adipokine and ghrelin levels on BMD and osteoporotic fractures through a meta-analysis.

**Data sources:**

Studies published till October 2020 in Medline, Embase, and the Cochrane Library were reviewed.

**Study selection:**

We included studies that measured at least one serum adipokine level and BMD or fracture risk in healthy individuals. We excluded studies with one or more of the following: patients less than 18 years old, patients with comorbidities, who had undergone metabolic treatment, obese patients, patients with high physical activities, and a study that did not distinguish sex or menopausal status.

**Data extraction:**

We extracted the data that include the correlation coefficient between adipokines (leptin, adiponectin, and resistin) and ghrelin and BMD, fracture risk by osteoporotic status from eligible studies.

**Data synthesis:**

A meta-analysis of the pooled correlations between adipokines and BMD was performed, demonstrating that the correlation between leptin and BMD was prominent in postmenopausal women. In most cases, adiponectin levels were inversely correlated with BMD. A meta-analysis was conducted by pooling the mean differences in adipokine levels according to the osteoporotic status. In postmenopausal women, significantly lower leptin (SMD = -0.88) and higher adiponectin (SMD = 0.94) levels were seen in the osteoporosis group than in the control group. By predicting fracture risk, higher leptin levels were associated with lower fracture risk (HR = 0.68), whereas higher adiponectin levels were associated with an increased fracture risk in men (HR = 1.94) and incident vertebral fracture in postmenopausal women (HR = 1.18).

**Conclusions:**

Serum adipokines levels can utilize to predict osteoporotic status and fracture risk of patients.

**Systematic review registration:**

https://www.crd.york.ac.uk/prospero/display_record.php?ID=CRD42021224855, identifier CRD42021224855.

## Introduction

Bones, the support system our body and protectors of internal organs, and adipose tissue, the largest endocrine tissue in the body, are closely related to nutrient metabolism and energy storage. Obesity plays a protective role in bone mineral density (BMD) (1, 2). However, a low body weight is a major risk factor for osteoporotic low-energy fractures (3, 4). Therefore, body mass index (BMI) obtained by diving body weight (in kilograms) by height (in meters) squared is included as a variable in the Fracture Risk Assessment Tool to calculate the fracture risk over 10 years (5). In contrast to previous reports, a high prevalence of obesity has been found in postmenopausal women with osteoporotic fractures (6).

Mesenchymal stem cells (MSCs) are pluripotent progenitor cells that mainly differentiate into adipocytes, osteoblasts, and chondroblasts (7). These three cell lineages differentiate from MSCs by common regulatory factors, such as hormones and cytokines, which determine their proliferation as well. Increased adiposity in the bone marrow of osteoporotic patients supports a link between bone and fat (8). Additionally, adipokines that include leptin, adiponectin, resistin, and visfatin are secreted from adipose tissue and affect bone metabolism, supporting the link between fat and bone (9, 10). Although not produced from adipose tissue, ghrelin, a type of growth hormone secretagogue, also affects lipid metabolism and regulates bone homeostasis (11, 12).

A meta-analysis was previously conducted on the correlation between blood concentrations of adipokines and ghrelin and BMD. The meta-analysis revealed that adiponectin had the inverse correlation with BMD (r = -0.14 to -0.4), independent of fat mass, BMI, and menopausal status. And leptin had the correlation with BMD (r = 0.1 to 0.33) (13). The relationship between the blood concentration of adipokines, bone density, and osteoporotic fractures has been studied extensively in the past 10 years. In particular, several studies have been conducted on the correlation between resistin and BMD measured at various sites, indicating that it has recently been in the spotlight as a biomarker for BMD (14–17). In addition, studies on the association between BMD and leptin or adiponectin have been conducted. Hence, we performed an updated meta-analysis on the impact of serum adipokines on BMD and osteoporotic fractures. According to our analysis, BMD was correlated with serum leptin level and was inversely correlated with serum adiponectin level in postmenopausal women. Furthermore, the fracture risk was predicted to be higher with a lower serum leptin level and higher serum adiponectin level. Serum adipokines levels can utilize to predict osteoporotic status and fracture risk of patients.

## Methods

This review was prospectively registered in PROSPERO (CRD42021224855) and followed the guidelines of the preferred reporting items for systematic reviews and meta-analyses.

### Search strategy

We searched the literature that was published from April 2010 to October 2020 using Medline, Embase, and the Cochrane Library. To identify studies that assessed the association between adipokines and BMD values, we searched the online databases with the following keywords: (‘adipokine’ OR ‘leptin’ OR ‘adiponectin’ OR ‘resistin’ OR ‘visfatin’ OR ‘ghrelin’) AND (‘bone density’ OR ‘osteoporosis’ OR ‘absorptiometry’ OR ‘fractures’). All searches were restricted to articles on human patients published in English.

### Inclusion criteria

Articles that met the following inclusion criteria were evaluated: 1) original studies that performed measurements on humans; 2) articles written in English; 3) studies that included measurement of BMD or fracture risk and at least one of the adipokines or ghrelin levels in serum; 4) studies that included BMD measured using dual-energy X-ray absorptiometry.

Studies with the following criteria were excluded: 1) patients less than 18 years old; 2) patients with comorbidities; 3) obese patients; 4) patients treated with metabolism medications (calcium and vitamin D excluded); 5) patients with high physical activities (such as an athlete); 6) did not distinguish sex or menopausal status in BMD (or fracture risk)-adipokine (or ghrelin) correlation.

### Data extraction

Two researchers independently checked the entire search, selection, and extraction processes. To resolve disagreements on matters related to the eligibility of studies or data extraction, a discussion was held between the two researchers or a counsel with a third researcher was included.

We filtered out conference abstracts, reviews, letters, and editorials from the list of studies. We then screened the remaining articles by confirming the title and abstract. After screening the articles, we examined the full text of the selected studies and categorized patients according to sex, menopausal status, assessed BMD site, and measured adipokines or ghrelin.

Finally, we extracted the following data from eligible studies: authors, year of publication, patients’ mean age, sex, menopausal status, osteoporotic status, fat mass, BMI, body weight, height, number of patients, method, site, score of BMD evaluation, method and serum level of each adipokines or ghrelin assessment, and correlation and multivariable regression of BMD with adipokines or ghrelin.

### Risk of bias in individual studies

We assessed the risk of bias for the individual cohort studies using the Newcastle-Ottawa scale (18). We used the modified version of the Newcastle-Ottawa scale to assess cross-sectional studies (19). The authors (SL, JHK) independently performed the risk of bias assessment in the included studies and confirmed the quality of evidence. The assessment results are presented in **Supplementary Tables S1**, **S2** .

### Publication bias

We determined whether there was a potential publication bias in the studies using funnel plots. Furthermore, we estimated the asymmetry of funnel plots using Egger’s regression test when a group included more than three studies.

### Certainty assessment

We assessed the certainty of evidence through the Grading of Recommendations, Assessment, Development, and Evaluations (GRADE) framework. This framework initiates with confirming the study design and then evaluating eight domains: risk of bias, indirectness, inconsistency, imprecision, publication bias, large effect, plausible confounding, and dose-response gradient. After assessing all the noted domains, the quality of evidence is classed as high, moderate, low, or very low (20).

### Statistical analyses

A meta-analysis of the pooled correlations between adipokines or ghrelin and BMD was conducted using the inverse of variance method. Furthermore, a random effects model was used in this study. Fisher’s z-transformation converted the non-adjusted (simple) correlation coefficients to calculate the pooled correlation coefficients (pooled r), 95% confidence interval (CI), and P value. We quantified statistical heterogeneity among the included studies by calculating the Q and I^2^ statistics (21). The pooling correlation meta-analysis and quality assessment of studies were executed using the ‘meta’ (22) and ‘dmetar’ (23) packages in R.

We also conducted a meta-analysis by pooling the mean differences in hormone levels according to osteoporotic status using the RevMan 5.0.1.8 software (Nordic Cochrane Center, Copenhagen, Denmark). Results reported in median and interquartile quartile range were converted to estimate the mean and standard deviation according to previously described methods (24). Standardized mean differences (SMD) were calculated for continuous outcome data (method of the inverse of the variance). Publication bias was determined to assess asymmetry using funnel plots.

## Results

### Selection and characteristics of studies

**Figure 1:**
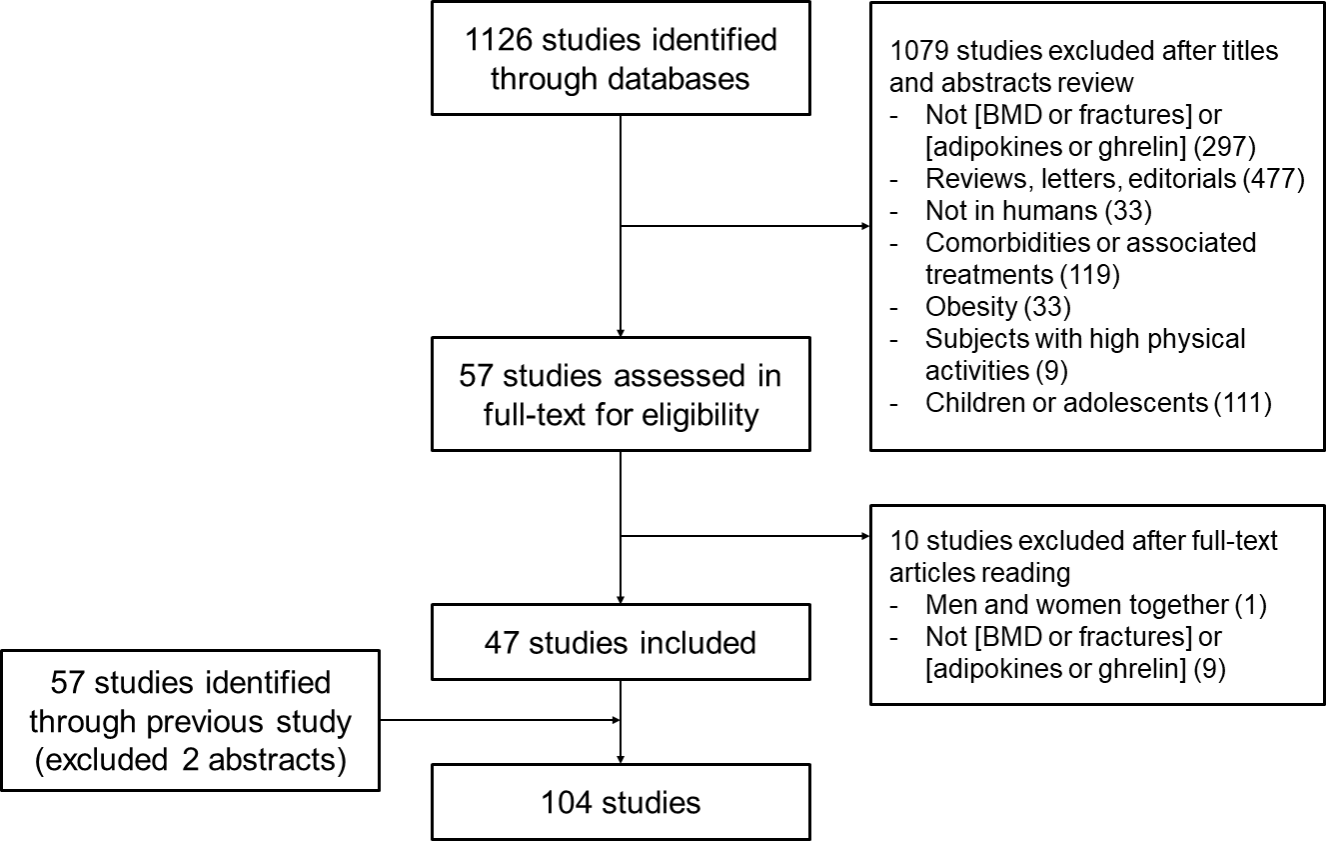
Flow chart depicting the search strategy employed to determine eligible studies.

The search process for the primary studies is shown in the flowchart in **Figure 1**. In the updated search, 1,126 studies, excluding duplicates, were identified through a database search. A total of 1079 studies were excluded from the assessment based on their title and abstract. The full text of the remaining 57 studies was assessed, and 10 studies were excluded because they did not meet the inclusion criteria; thus, 47 studies were selected for the meta-analysis. Of the 59 studies included in the previous meta-analysis, 57 were included in our study, excluding two abstracts (13). Two abstracts were excluded because they overlapped with the published literature or were inaccessible. Finally, 104 studies were included in this study (14–17, 25–124). The pooled correlation analysis included 11,960 participants (4,790 men, 1,392 premenopausal women, and 5,778 postmenopausal women) across 48 studies. The mean age of participants was 56.6 years for men, 36.3 years for premenopausal women, and 62.8 years for postmenopausal women. The mean BMI was 25.85 kg/m^2^, 21.36 kg/m^2^, and 25.12 kg/m^2^ for men, premenopausal women, and postmenopausal women, respectively. The mean BMD in lumbar spine site was 1.12 g/cm^2^, 1.11 g/cm^2^, and 0.92 g/cm^2^ for men, premenopausal women, and postmenopausal women, respectively. The mean BMD in femoral neck site was 0.91 g/cm^2^, 1.02 g/cm^2^, and 0.77 g/cm^2^ for men, premenopausal women, and postmenopausal women, respectively. The mean BMD in total body was 1.04 g/cm^2^, 1.15 g/cm^2^, and 1.00 g/cm^2^ for men, premenopausal women, and postmenopausal women, respectively.

### Correlations between serum adipokine and ghrelin levels and BMD

We conducted a pooled correlation analysis on the selected studies according to sex, menopausal status, and BMD site (**Table 1**). The funnel plot for each adipokine, ghrelin, and BMD site is shown in **Supplementary Figure S1**. To verify the symmetry of each plot, we performed Egger’s test (**Table 2**). A publication bias was found in the correlation between total hip BMD and leptin levels in men; however, no publication bias was detected in other studies. The certainty of the evidence was determined by assessing the eight domains for the outcome of correlations of adipokine levels and BMD. Because all included studies are observational studies, GRADE defaults to low, and some are downgraded to very low due to the risk of bias and inconsistency (**Supplementary Table S3**).

**Table 1:** Pooled correlations between adipokines or ghrelin level and BMD according to sex and menopausal status.

Group	Adipokine/ghrelin	BMD site	No. of patients	Heterogeneity		Random effects model			Studies
				I^2^	p	r	95% CI	p	
**Men**	Leptin	Lumbar spine	2266	78	< 0.001	0.05	-0.06, 0.15	0.38	(14, 43, 46, 72, 76, 89, 94, 111)
		Total hip	916	30	0.22	0.12	0.04, 0.2	0.004	(14, 72, 94, 111, 123)
		Femoral neck	2146	76	< 0.001	0.11	0.01, 0.21	0.03	(14, 43, 46, 72, 76, 89, 91, 123)
		Total body	1958	36	0.16	0.09	0.02, 0.15	0.009	(14, 72, 76, 82, 94, 123)
	Adiponectin	Lumbar spine	1201	0	0.61	-0.19	-0.25, -0.14	< 0.001	(33, 50, 72, 79, 89, 94)
		Total hip	1029	56	0.08	-0.17	-0.27, -0.08	< 0.001	(50, 72, 79, 94)
		Femoral neck	528	31	0.23	-0.16	-0.26, -0.06	0.003	(33, 50, 72, 89)
		Total body	1029	94	< 0.001	-0.35	-0.56, -0.1	0.007	(50, 72, 79, 94)
	Resistin	Lumbar spine	561	31	0.22	-0.1	-0.21, 0.01	0.07	(14, 72, 89, 94)
		Total hip	249	0	0.58	-0.08	-0.21, 0.04	0.2	(14, 72)
		Femoral neck	249	0	0.93	-0.03	-0.16, 0.09	0.59	(14, 72)
		Total body	249	18	0.27	-0.09	-0.26, 0.08	0.28	(14, 72)
	Ghrelin	Lumbar spine	821	0	0.56	-0.05	-0.12, 0.02	0.19	(50, 77, 89, 116)
		Total hip	741	80	0.006	0.05	-0.15, 0.25	0.6	(50, 77, 116)
		Femoral neck	742	85	0.001	0.09	-0.14, 0.31	0.45	(50, 89, 116)
		Total body	216	0	0.34	0.13	-0.01, 0.26	0.06	(50, 77)
**Premenopausal women**	Leptin	Lumbar spine	853	4	0.4	0.08	0.01, 0.15	0.03	(15, 43, 55, 59, 60, 67, 92, 111)
		Total hip	320	0	0.84	0.28	0.17, 0.37	< 0.001	(15, 67, 111)
		Femoral neck	669	45	0.1	0.09	-0.02, 0.19	0.12	(15, 43, 59, 60, 67, 92)
		Total body	624	0	0.99	0.19	0.11, 0.26	< 0.001	(15, 55, 59, 60, 68, 92)
	Adiponectin	Lumbar spine	336	57	0.1	-0.07	-0.25, 0.11	0.45	(15, 44, 60)
		Total hip	38	NA	NA	-0.13	-0.43, 0.2	0.44	(15)
		Femoral neck	336	0	0.47	-0.13	-0.23, -0.02	0.02	(15, 44, 60)
		Total body	240	22	0.28	-0.25	-0.38, -0.1	< 0.001	(15, 60, 68)
	Resistin	Lumbar spine	38	NA	NA	-0.05	-0.36, 0.27	0.77	(15)
		Total hip	38	NA	NA	-0.25	-0.53, 0.08	0.13	(15)
		Femoral neck	38	NA	NA	-0.21	-0.5, 0.12	0.21	(15)
		Total body	38	NA	NA	-0.15	-0.45, 0.18	0.37	(15)
**Postmenopausal women**	Leptin	Lumbar spine	3456	84	< 0.001	0.18	0.09, 0.27	< 0.001	(15, 16, 36, 38, 43, 46, 47, 55, 62, 67, 84, 85, 87, 88, 92, 98, 100, 111, 113, 118, 120)
		Total hip	2159	35	0.13	0.29	0.23, 0.34	< 0.001	(15, 16, 48, 67, 85, 98, 111, 120, 123, 124)
		Femoral neck	1965	42	0.03	0.22	0.17, 0.28	< 0.001	(15, 16, 36, 38, 43, 46, 62, 67, 84, 88, 92, 98, 100, 106, 114, 118, 123, 124)
		Total body	1625	72	< 0.001	0.26	0.16, 0.35	< 0.001	(15, 36, 47, 55, 62, 78, 92, 100, 106, 114, 118, 120, 123, 124)
	Adiponectin	Lumbar spine	2850	9	0.36	-0.16	-0.2, -0.12	< 0.001	(15, 16, 30, 62, 66, 79, 84, 85, 90, 110, 112, 120)
		Total hip	2053	0	0.46	-0.23	-0.27, -0.18	< 0.001	(15, 16, 79, 85, 90, 95, 120, 124)
		Femoral neck	1024	52	0.04	-0.23	-0.32, -0.13	< 0.001	(15, 16, 62, 66, 84, 95, 112, 124)
		Total body	972	46	0.12	-0.17	-0.27, -0.07	0.001	(15, 62, 79, 120, 124)
	Resistin	Lumbar spine	678	88	< 0.001	-0.03	-0.26, 0.2	0.8	(15–17, 120)
		Total hip	518	48	0.15	0.07	-0.07, 0.2	0.33	(15, 16, 120)
		Femoral neck	342	93	< 0.001	-0.02	-0.43, 0.39	0.91	(15–17)
		Total body	391	83	0.01	0.12	-0.23, 0.44	0.52	(15, 120)
	Ghrelin	Lumbar spine	581	39	0.19	-0.07	-0.21, 0.06	0.29	(62, 77, 116)
		Total hip	493	0	0.7	-0.04	-0.12, 0.05	0.44	(77, 116)
		Femoral neck	540	35	0.22	-0.07	-0.2, 0.06	0.28	(62, 116)
		Total body	129	0	0.42	-0.05	-0.04, 0.17	0.55	(62, 77)

NA, not applicable.

**Table 2:** Summarized results of the Egger’s test.

Group/Adipokine	BMD site	Bias	p	Studies
Men				
Leptin	Lumbar spine	1.614	0.344	(14, 43, 46, 72, 76, 89, 94, 111)
	Total hip	2.973	0.006	(14, 72, 94, 111, 123)
	Femoral neck	1.125	0.478	(14, 43, 46, 72, 76, 89, 91, 123)
	Total body	0.734	0.555	(14, 72, 76, 82, 94, 123)
Adiponectin	Lumbar spine	0.414	0.767	(33, 50, 72, 79, 89, 94)
	Total hip	0.588	0.905	(50, 72, 79, 94)
	Femoral neck	-0.787	0.846	(33, 50, 72, 89)
	Total body	-12.946	0.236	(50, 72, 79, 94)
Resistin	Lumbar spine	-2.728	0.072	(14, 72, 89, 94)
	Total hip	NA	NA	(14, 72)
	Femoral neck	NA	NA	(14, 72)
	Total body	NA	NA	(14, 72)
Ghrelin	Lumbar spine	1.376	0.215	(50, 77, 89, 116)
	Total hip	3.603	0.444	(50, 77, 116)
	Femoral neck	4.572	0.355	(50, 89, 116)
	Total body	NA	NA	(50, 77)
Premenopausal women				
Leptin	Lumbar spine	-1.436	0.308	(15, 43, 55, 59, 60, 67, 92, 111)
	Total hip	-0.459	0.759	(15, 67, 111)
	Femoral neck	-1.057	0.653	(15, 43, 59, 60, 67, 92)
	Total body	-0.601	0.201	(15, 55, 59, 60, 68, 92)
Adiponectin	Lumbar spine	0.980	0.840	(15, 44, 60)
	Total hip	NA	NA	(15)
	Femoral neck	0.361	0.897	(15, 44, 60)
	Total body	4.052	0.216	(15, 60, 68)
Resistin	Lumbar spine	NA	NA	(15)
	Total hip	NA	NA	(15)
	Femoral neck	NA	NA	(15)
	Total body	NA	NA	(15)
Postmenopausal women				
Leptin	Lumbar spine	0.055	0.960	(15, 16, 36, 38, 43, 46, 47, 55, 62, 67, 84, 85, 87, 88, 92, 98, 100, 111, 113, 118, 120)
	Total hip	-0.208	0.817	(15, 16, 48, 67, 85, 98, 111, 120, 123, 124)
	Femoral neck	-0.296	0.804	(15, 16, 36, 38, 43, 46, 62, 67, 84, 88, 92, 98, 100, 106, 114, 118, 123, 124)
	Total body	1.343	0.284	(15, 36, 47, 55, 62, 78, 92, 100, 106, 114, 118, 120, 123, 124)
Adiponectin	Lumbar spine	-0.233	0.758	(15, 16, 30, 62, 66, 79, 84, 85, 90, 110, 112, 120)
	Total hip	-0.898	0.239	(15, 16, 79, 85, 90, 95, 120, 124)
	Femoral neck	-1.843	0.234	(15, 16, 62, 66, 84, 95, 112, 124)
	Total body	-0.854	0.604	(15, 62, 79, 120, 124)
Resistin	Lumbar spine	2.552	0.681	(15–17, 120)
	Total hip	3.208	0.074	(15, 16, 120)
	Femoral neck	9.979	0.523	(15–17)
	Total body	NA	NA	(15, 120)
Ghrelin	Lumbar spine	-1.443	0.552	(62, 77, 116)
	Total hip	NA	NA	(77, 116)
	Femoral neck	NA	NA	(62, 116)
	Total body	NA	NA	(62, 77)

NA, not applicable.

Over the past decade, many studies have been published on the correlation between adipokine levels and BMD. Several studies have also examined the correlation between leptin and BMD in postmenopausal women, as well as the impact of pre- and post- menopause on adiponectin. A decade ago, only few studies on resistin were conducted; however, since then several new studies for all groups have been performed. Although there are new findings for ghrelin, these could not be used for the meta-analysis because the correlation was not analyzed (**Supplementary Figure S2**).

In postmenopausal women, leptin level was positively correlated with BMD at the lumbar spine, total hip, femoral neck, and total body (r = 0.18 to 0.29). In addition, the correlation was more robust in postmenopausal women than in other cohorts. In premenopausal women, the correlation was significant at three the sites other than the femoral neck site (r = 0.08 to 0.28). Although the leptin level and BMD correlation at total hip, femoral neck, and total body sites was significant in men (r = 0.09 to 0.12), the correlation coefficients were slightly lower than those in the other two groups (14–16, 36, 38, 43, 46–48, 55, 59, 60, 62, 67, 68, 72, 76, 78, 82, 84, 85, 87–89, 91, 92, 94, 98, 100, 106, 111, 113, 114, 118, 120, 123, 124).

In men and postmenopausal women, adiponectin and BMD at all sites showed a significant inverse correlation (men: r = -0.16 to -0.35, postmenopausal women: r = -0.16 to -0.23). In premenopausal women, adiponectin and BMD correlation was only significant at the femoral neck (r = -0.13) and total body (r = -0.25) sites (15, 16, 30, 33, 44, 50, 60, 62, 66, 68, 72, 79, 84, 85, 89, 90, 94, 95, 110, 112, 120, 124).

Based on the above results, correlations between resistin (14–17, 72, 89, 94, 120) or ghrelin levels (50, 62, 77, 89, 116) and BMD were not statistically significant at any sites.

As the correlations between adipokines and BMD adjusted by body weight or BMI were reduced compared to non-adjusted correlations (125), we examined the data of both non-adjusted and adjusted correlations using body weight, BMI, or fat mass (**Table 3**). The correlations between leptin and BMD were generally weakened or became insignificant after adjustment for anthropometric measures, and these phenomena were distinct in postmenopausal women as well as men. However, even though the inverse correlation between adiponectin and BMD weakened even after adjustment, most studies still revealed that the correlation was significant. Although the correlations were weakened for resistin, they remained significant in postmenopausal women (lumbar spine and femoral neck) (120). Studies examining the correlation between visfatin and BMD showed a significant correlation with only total hip in men (r = 0.18) and lumbar spine in postmenopausal women (r = 0.113). However, after adjustment for anthropometric measures, all correlations of visfatin and BMD weakened (94, 112, 120).

**Table 3:** Non-adjusted and adjusted correlations between adipokines or ghrelin levels and BMD according to sex and menopausal status.

Group/Adipokine	BMD site	Studies	No. of patients	Without adjustment		Adjusted for fat-related variables	
				r	p	r	p
Men							
Leptin	Lumbar spine	Thomas, 2001 (111)	343	-0.12	<0.05	-0.09^a^	NS
		Oh, 2005 (89)	80	-0.08	0.489	-0.24^b^	0.039
		Peng, 2008 (94)	232	0.13	NS	0.01^c^	NS
		Dennison, 2004 (46)	219	0.27	<0.001	0.10^d^	NS
	Total hip	Thomas, 2001 (111)	343	0.05	NS	-0.15^a^	<0.01
		Peng, 2008 (94)	232	0.13	NS	0.05^c^	NS
		Zoico, 2003 (123)	92	0.23	<0.05	0.13^a^	0.236
	Femoral neck	Zoico, 2003 (123)	92	0.25	<0.05	0.13^a^	0.21
		Dennison, 2004 (46)	219	0.30	<0.001	0.04^d^	NS
	Total body	Peng, 2008 (94)	232	-0.01	NS	-0.05^c^	NS
		Morberg, 2003 (82)	317	0.17	<0.05	-0.19^g^	<0.01
		Zoico, 2003 (123)	92	0.19	0.064	0.25^a^	<0.05
Adiponectin	Total hip	Peng, 2008 (94)	232	-0.26	<0.05	-0.14^c^	<0.05
	Femoral neck	Basurto, 2009 (33)	92	-0.24	<0.001	-0.09^e^	NS
	Total body	Peng, 2008 (94)	232	-0.21	<0.05	-0.15^c^	<0.05
Resistin	Lumbar spine	Oh, 2005 (89)	80	-0.24	0.05	-0.31^b^	0.011
		Peng, 2008 (94)	232	-0.02	NS	0.01^c^	NS
	Total hip	Peng, 2008 (94)	232	-0.08	NS	-0.04^c^	NS
	Total body	Peng, 2008 (94)	232	-0.05	NS	-0.09^c^	NS
Visfatin	Lumbar spine	Peng, 2008 (94)	232	0.08	NS	0.05^c^	NS
	Total hip	Peng, 2008 (94)	232	0.18	<0.05	0.14^c^	NS
	Total body	Peng, 2008 (94)	232	0.02	NS	0.01^c^	NS
Ghrelin	Femoral neck	Gonnelli, 2008 (50)	137	0.25	<0.01	0.20^f^	<0.05
Premenopausal women							
Leptin	Lumbar spine	Thomas, 2001 (111)	137	0.05	NS	-0.01^a^	NS
	Total hip	Thomas, 2001 (111)	137	0.31	<0.001	-0.04^a^	NS
Postmenopausal women							
Leptin	Lumbar spine	Thomas, 2001 (111)	165	0.25	<0.01	0.08^a^	NS
		Dennison, 2004 (46)	172	0.36	<0.001	0.14^d^	NS
		Zhang, 2010 (120)	336	0.066	NS	-0.03^c^	NS
	Total hip	Zoico, 2003 (123)	171	0.34	<0.001	0.15^a^	<0.05
		Thomas, 2001 (111)	165	0.44	<0.001	-0.01^a^	NS
		Zhang, 2010 (120)	336	0.162	<0.05	0.06^c^	NS
	Femoral neck	Zoico, 2003 (123)	171	0.33	<0.001	0.16^a^	<0.05
		Dennison, 2004 (46)	172	0.35	<0.001	0.10^d^	NS
	Total body	Zoico, 2003 (123)	171	0.33	<0.001	0.30^a^	<0.001
		Zhang, 2010 (120)	336	0.064	NS	0.02^c^	NS
Adiponectin	Lumbar spine	Tohidi, 2012 (112)	382	-0.19	0.0001	-0.09^h^	0.097
		Zhang, 2010 (120)	336	-0.208	<0.05	-0.14^c^	NS
	Total hip	Zoico, 2008 (124)	36	-0.46	<0.001	-0.36^a^	<0.05
		Zhang, 2010 (120)	336	-0.228	<0.05	-0.15^c^	<0.05
	Femoral neck	Zoico, 2008 (124)	36	-0.45	<0.001	-0.36^a^	<0.05
		Tohidi, 2012 (112)	382	-0.14	0.008	-0.03^h^	0.56
	Total body	Zoico, 2008 (124)	36	-0.52	<0.001	-0.42^a^	<0.001
		Zhang, 2010 (120)	336	-0.228	<0.05	-0.13^c^	<0.05
Resistin	Lumbar spine	Tariq, 2020 (17)	160	-0.359	<0.001	-0.26^i^	0.001
		Zhang, 2010 (120)	336	-0.043	NS	-0.04^c^	NS
	Total hip	Zhang, 2010 (120)	336	-0.022	NS	-0.02^c^	NS
	Femoral neck	Tariq, 2020 (17)	160	-0.4	<0.001	-0.26^i^	0.001
	Total body	Zhang, 2010 (120)	336	-0.043	NS	-0.03^c^	NS
Visfatin	Lumbar spine	Tohidi, 2012 (112)	382	0.113	0.043	0.07^h^	0.223
		Zhang, 2010 (120)	336	-0.05	NS	-0.05^c^	NS
	Total hip	Zhang, 2010 (120)	336	-0.027	NS	-0.02^c^	NS
	Femoral neck	Tohidi, 2012 (112)	382	0.084	NS	0.03^h^	0.581
	Total body	Zhang, 2010 (120)	336	-0.054	NS	-0.05^c^	NS

NS, not significant.

Adjustment for anthropometric measures:

a Fat mass;

b age, BMI;

c age, fat mass;

d age, alcohol, tobacco, activities, calcium intake, osteoarthritis, BMI;

e BMI;

f age, BMI, calcium intake;

g body weight;

h age, weight;

i age, hip girth, waist girth, waist to hip (W/H) ratio, weight, height, and BMI.

We further performed subgroup analysis by geographical populations. In men, pooled correlation coefficients (r) of leptin with BMD were higher in Europe (r = 0.12 to 0.27) populations than in other regions (r = -0.12 to 0.11). Correlations of adiponectin with BMD differed by region but did not appear consistently. Correlations of resistin and ghrelin with BMD were slightly stronger in Europe (resistin: r = -0.05 to -0.31; ghrelin: r = 0.04 to 0.25) than in other areas (resistin: r = -0.03 to -0.08; ghrelin: r = -0.08 to 0.12) (**Supplementary Table S4**). In premenopausal women, the correlation between entire groups did not appear tendency by region (**Supplementary Table S5**). In postmenopausal women, correlations of leptin with BMD were weaker in Asia (r = 0.07 to 0.25) than in other regions (r = 0.14 to 0.44). Correlations of adiponectin with BMD were similar in all areas. Interestingly, correlations of resistin with BMD were positive in Europe (r = 0.15 to 0.31) and were not in Asia (r = -0.02 to -0.40). Correlations of ghrelin with BMD were more robust in Europe (r = -0.10 to -0.22) than in other regions (r = -0.05 to 0.05) (**Supplementary Table S6**).

### Associations between adipokine and ghrelin levels and BMD

Regression analyses between BMD and adipokines or ghrelin levels were performed in 42 studies (**Supplementary Tables S7, S8**) (15–17, 26, 28, 29, 33, 35–37, 43, 45, 48, 59–62, 64, 66, 68, 70, 72, 76, 77, 79, 82, 89, 92, 94, 99, 107, 110, 112, 114–118, 120, 122–124).

Multiple regression analyses were performed to determine the variable, including adiponectin, which significantly correlated with the BMD value. A significant inverse correlation between BMD and adiponectin levels was found in 10 of the 16 studies (28, 60, 62, 64, 66, 68, 94, 117, 120, 124). One study that included men revealed that there was a inverse association between lumbar spine (β = -0.163), total hip (β = -0.148), and total body (β = -0.178) BMD and adiponectin levels (94). Three studies that included premenopausal women revealed a inverse association between lumbar spine (β = -0.283; -0.01), femoral neck (β = -0.01), and total body (β = -0.152; -0.01; -0.26) and adiponectin levels (60, 64, 68). Six studies that included postmenopausal women, revealed a inverse association between lumbar spine (β = -0.006; -0.103; B = -2.684), femoral neck (β = -0.27; -0.047; -0.445), total hip (β = -0.112; B = -2.247), total forearm (β = -0.125; B = -2.167), and total body (β = -0.105, -0.385, B = -2.54), and adiponectin level (28, 62, 66, 117, 120, 124). However, no such association was found in six studies (16, 33, 70, 72, 110, 112).

The results of studies examining the association between leptin and BMD are heterogeneous. In men, only one study revealed a positive association (total hip: β = 0.097) (99), and all other study results were not significant or demonstrated a inverse association (43, 45, 76, 82, 89, 94, 107, 123). For women, only two studies with premenopausal women (37, 59) and four studies with postmenopausal women (36, 62, 118, 123) revealed a positive association, whereas the others revealed no significance or a inverse association (16, 28, 35, 43, 48, 60, 70, 92, 99, 114, 117, 122, 124). By adjusting leptin levels by body composition-related variables, the association between leptin and BMD was either weakened, disappeared, or even inverted.

Three studies investigated the association between resistin and BMD (15, 17, 28), and only one found an association (total body BMD of postmenopausal women: β = 0.31) (15). Of the three studies (26, 62, 77) that examined the association between ghrelin and BMD, only one found an association (total hip BMD of young women: β = -0.31) (62).

Collectively, the impact of plasma adipokines or ghrelin levels on BMD would be weak and might be confounded by other body composition parameters.

### Associations between adipokine and ghrelin levels and BMD changes

The potential of adipokines or ghrelin to predict BMD changes was assessed in five cohort studies (29, 32, 45, 49, 63).

Araneta et al. reported that adiponectin was not associated with bone loss in men and postmenopausal women (29). According to Barbour et al., adiponectin was associated with hip BMD changes in the highest tertile women (Mean annualized % change = -0.67%) compared to in the lowest tertile (Mean annualized % change = -0.43%) after adjusting for age, race, BMI, diabetes, baseline hip aBMD, and weight change. Leptin was not associated with BMD changes in either men or women (32). Crabbe et al. investigated the correlation between leptin and total hip and forearm BMD changes in older men; however, their results were not statistically significant (45). Fuggle et al. investigated the association between lumbar spine and femoral neck BMD changes with leptin and adiponectin, but they found no association (49). Jürimäe et al. investigated the association between BMD changes and adipokine levels in postmenopausal women, and found a positive association between total body (β = 0.001) and femoral neck (β = 0.001) BMD reduction and leptin, and an inverse association between lumbar spine BMD reduction (β = -0.002) and adiponectin (63).

Based on these results, plasma adipokines or ghrelin levels had a weak or no association with the prediction of BMD changes.

### Differences in adipokines or ghrelin levels in patients according to osteoporosis status

**Figure 2:**
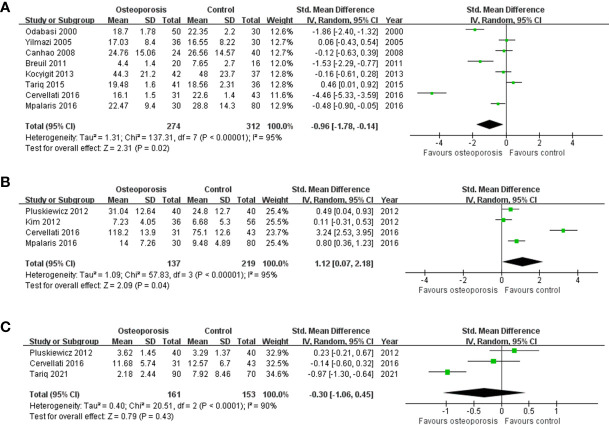
Forest plot depicting the differences in adipokine levels according to osteoporosis status in postmenopausal women **(A)** Leptin, **(B)** adiponectin, and **(C)** resistin.

A total of 12 studies on the level of adipokines or ghrelin according to the diagnosis of osteoporosis were included (**Table 4**) (16, 38, 41, 50, 66, 69, 84, 87, 95, 108, 109, 119). The meta-analysis results for leptin levels in postmenopausal women are shown in **Figure 2A**. Nine studies for leptin involving 757 participants, revealed a high heterogeneity (P < 0.001, I^2 ^= 94%). In postmenopausal women, leptin levels were significantly lower in the osteoporosis group than in the normal BMD group (SMD = -0.88, 95% CI = -1.55, -0.21, P = 0.01). There were two studies on leptin levels according to the presence or absence of osteoporosis in men; however, there was no significant difference between the two groups (SMD = -0.10, 95% CI = -0.39, 0.20, P = 0.52; I^2 ^= 0%, P = 0.72). The five studies on adiponectin involved 527 postmenopausal participants, and revealed a significantly higher adiponectin level in osteoporotic women with high heterogeneity (SMD = 0.94, 95% CI = 0.17, 1.71, P = 0.02; I^2 ^= 95%, P < 0.001) (**Figure 2B**). As shown in **Figure 2C**, three studies on resistin involved 314 postmenopausal women. No significant difference in resistin levels was observed between the osteoporotic and control groups in postmenopausal women (SMD = -0.30, 95% CI = -1.06, 0.45, P = 0.43; I^2 ^= 90%, P < 0.001). All adipokine levels in premenopausal women and adiponectin or resistin levels in men were insufficient for meta-analysis. For other adipokines or ghrelin, insufficient data were available for a meta-analysis.

**Table 4:** Differences in adipokines or ghrelin levels according to osteoporosis status.

Studies	Group	Adipokine/ Ghrelin	Osteoporosis		Normal BMD		
			No.	Mean ± SD	No.	Mean ± SD	p
Odabasi, 2000 (87)	Postmenopausal	Leptin (ng/ml)	50	18.7±1.79	30	22.35±2.2	0.103
Yilmazi, 2005 (119)	Postmenopausal	Leptin (ng/ml)	36	17.03±8.4	30	16.55±8.22	0.15
Canhao, 2008 (41)	Women > 50 yr	Leptin (ng/ml)	24	24.76±15.06	40	26.56±14.57	NS
Kocyigit, 2013 (69)	Postmenopausal	Leptin (ng/ml)	42	44.3±21.2	37	48±23.7	NS
Tariq, 2015 (109)	Postmenopausal	Leptin (ng/ml)	41	19.48±1.6	36	18.56±2.31	NS
Breuil, 2011 (38)	Postmenopausal	Leptin (ng/ml)	20	4.4±1.4	16	7.65±2.7	0.002
Mpalaris, 2016 (84)	Postmenopausal	Leptin (ng/ml)	30	22.47±9.4	80	28.8±14.3	< 0.001
Cervellati, 2016 (16)	Postmenopausal	Leptin (ng/ml)	31	16.1±1.5	43	22.6±1.4	<0.05
Tanna, 2017 (108)	Postmenopausal	Leptin (ng/ml)	83	22±20.3	88	29.6±20.2	<0.01
Papadopoulau, 2004 (91)	Men	Leptin (ng/ml)	44	12.7±11.2	319	14.1±12	NS
Canhao, 2008 (41)	Men	Leptin (ng/ml)	10	9.72±7.63	19	9.48±7.13	NS
Cervellati, 2016 (16)	Postmenopausal	Adiponectin (µg/ml)	31	118.2±13.9	43	75.1±12.6	<0.05
Kim, 2012 (66)	Postmenopausal	Adiponectin (µg/ml)	36	7.23±4.05	56	6.68±5.3	NS
Mpalaris, 2016 (84)	Postmenopausal	Adiponectin (µg/ml)	30	14±7.26	80	9.48±4.89	< 0.001
Pluskiewicz, 2012 (95)	Postmenopausal	Adiponectin (µg/ml)	40	31.04±12.64	40	24.81±12.7	<0.05
Tanna, 2017 (108)	Postmenopausal	Adiponectin (µg/ml)	83	20.2±9.2	88	17.5±8.6	<0.05
Gonnelli, 2008 (50)	Men	Adiponectin (µg/ml)	25	10.1±5.3	47	11.3±3.8	NS
Gonnelli, 2008 (50)	Men	Ghrelin (pg/ml)	25	757.5±92.4	47	853.6±136.8	NS
Mpalaris, 2016 (84)	Postmenopausal	Ghrelin (pg/ml)	30	322.5±172.81	80	309.27±140.89	NS
Tariq, 2021 (17)	Postmenopausal	Resistin (ng/ml)	90	2.18±2.44	70	7.92±8.46	<0.001
Cervellati, 2016 (16)	Postmenopausal	Resistin (ng/ml)	31	11.68±5.74	43	12.57±6.7	NS
Pluskiewicz, 2012 (95)	Postmenopausal	Resistin (ng/ml)	40	3.62±1.45	40	3.29±1.37	NS

NS, not significant.

### Correlation between adipokines or ghrelin levels and fragile osteoporotic bone fracture

Three studies reported an association between adipokines or ghrelin levels and the prevalence of vertebral fractures. Prevalent vertebral fracture was observed in 15–35% of participants (85, 108, 118). Two studies demonstrated an inconsistent association between leptin or adiponectin levels and prevalence of vertebral fracture, and one of the two studies was included in a previous meta-analysis. No data were available for other adipokines or ghrelin. Leptin level was positively correlated with the percentage of fat mass. Furthermore, only leptin levels predicted the presence of vertebral fractures in the logistic regression model (odds ratio [OR] = 0.642, 95% CI = 0.429, 0.960; p = 0.031) (118). By contrast, serum leptin level was not associated with fracture risk (OR = 1.006, 95% CI = 0.989, 1.023; p = 0.495) adjusted for age, years since menopause, fat-related parameters, and lifestyle variables (108). The pooled OR for leptin was 0.84 (95% CI = 0.55, 1.30; p = 0.43) (108, 118). Serum adiponectin level was associated with the above-adjusted fracture risk but was not statistically significant (OR = 1.034, 95% CI = 0.998, 1.071; p = 0.06) (108).

A total of six prospective cohort studies reported the association between adipokines and incident fractures (29, 31, 58, 79, 85, 102), and three new articles were included. Three studies reported a relationship between leptin and fracture outcomes (31, 85, 102). Two studies showed inconsistent fracture risk in postmenopausal women; one study with men found no association with fracture risk according to serum leptin levels (31, 85). In a cohort study with an average follow up of 6.5 years, higher leptin levels resulted in lower fracture rates based on an unadjusted model in postmenopausal women (high tertile hazard ratio [HR] = 0.68, middle tertile HR = 0.74; p = 0.009); however, in the adjusted model for age, race, and BMI, the association of leptin levels and fracture rates was attenuated (high tertile HR = 0.98, middle tertile HR = 0.86; p = 0.794) (31). Nakamura et al. showed that lower serum leptin levels were a significant risk factor for incident long-bone fractures (HR = 0.70; 95% CI = 0.50, 0.96) adjusted for age, body weight, hip BMD, prevalent fracture, osteoporosis treatment, serum albumin, calcium, and adiponectin (85). In a study that analyzed men and women together, the high tertile group with serum leptin levels showed lower fracture risk than the low tertile groups after adjusting for factors (age, sex, menopausal status, body weight, social status, smoking, alcohol consumption, physical activity, diabetes, and creatinine) (102). The HR was 0.25 (95% CI = 0.09, 0.74; p = 0.01 for trend).

For adiponectin, five studies reported a relationship between adiponectin and fracture outcomes (29, 31, 58, 79, 85). Three of the four studies found an association with fracture risk in men (29, 31, 58, 79), and two studies showed inconsistent fracture risk in postmenopausal women according to serum adiponectin levels (31, 85). Michaelsson et al. found that despite the inverse association between adiponectin and BMD, adiponectin did not increase fracture risk in men (adjusted HR = 0.97, 95% CI = 0.86, 1.10; p > 0.05) (79). A community-based longitudinal study followed up fracture data from 277 of 284 men with serial measures, where 21 (7.6%) had at least one vertebral fracture (29). Adiponectin was independently associated with vertebral fractures only in men. The adjusted OR was 1.13 (95% CI: 1.08, 1.23; p = 0.009). Fracture data from 251 of the 261 women with serial measures, revealed that 48 (19.1%) women had a vertebral fracture but no association with adiponectin. Based on a 7.4-years (average, 5.2 years) follow up with the MrOS Sweden cohort of 999 men (58), 150 men (15%) had fractures, with spine fracture being the most common. Adiponectin was associated with a significantly higher incidence of fracture in participants (HR/SD = 1.46; 95% CI = 1.23, 1.72), which was maintained after multivariate adjustment variables for age, time, total hip BMD, general health, and previous fracture (HR = 1.30; 95% CI = 1.09, 1.55). Barbour et al. (31) reported that the fracture rates per 1000 person-years were 27.5 and 14.0 for women and men, respectively, based on a mean follow up of 6.5 years. Adiponectin was significantly associated with fracture risk in men with the highest adiponectin level quartile compared to the lowest quartile (HR = 1.94; 95% CI = 1.20, 3.16) adjusted for age, race, BMI, education, weight change, and total hip BMD. However, no association was found between adiponectin levels and fracture risk in women (HR = 0.98; 95% CI = 0.67, 1.43). Nakamura et al. reported that higher serum adiponectin levels were a significant independent risk factor for incident vertebral fractures in postmenopausal women. The HR of serum adiponectin was 1.18 (95% CI 1.02–1.37, after adjusting for age, body weight, lumbar BMD, prevalent fracture, osteoporosis treatment, serum albumin, calcium, and leptin) (85).

## Discussion

We performed an updated meta-analysis on the effects of serum adipokines or ghrelin levels on BMD and fracture risk in healthy adults. Our meta-analysis revealed that postmenopausal women with osteoporosis had significantly lower serum leptin concentrations and higher serum adiponectin concentrations than those in postmenopausal women with normal BMD. Accordingly, the osteoporotic status can be predicted using serum concentrations of leptin and adiponectin in postmenopausal women. In a previous meta-analysis, serum adiponectin levels were not significantly associated with femoral neck BMD in postmenopausal women; however, in this study, BMD values from the lumbar spine, total hip, femoral neck, and total body in postmenopausal women showed a positive correlation with leptin level and a inverse correlation with adiponectin level, which was statistically significant. The correlations between serum leptin or adiponectin concentrations and BMD values from various sites in men and premenopausal women were almost similar to those of the previous meta-analysis, which demonstrated that femoral neck BMD in men and leptin or adiponectin showed significant correlations, and total body BMD in premenopausal women was significantly correlated with adiponectin level. After adjusting for anthropometric measures, the adiponectin concentrations showed a significant correlation with the BMD value; however, leptin concentrations were not significantly correlated most studies. Although serum resistin concentration did not significantly correlate with the BMD values in the pooled analysis, two studies demonstrated a significant inverse correlation with the lumbar spine BMD values in both postmenopausal women and men, even after adjusting for anthropometric measures (17, 89). Although leptin levels and prevalent vertebral fractures in one study were previously reported to be significant (118), the OR value in the pooled analysis with another study was not significant (108, 118).

Among the 39 pooled analyses listed in **Table 1**, 13 studies showed high heterogeneity. We attempted to reduce this heterogeneity by reducing the influence of confounders to more accurately determine the effect of adipokines on bone. To rule out the effects of comorbidities or treatments, we only included studies in which healthy participants were enrolled. To diminish this confounding effect, a pooled analysis based on adjusting for anthropometric measures is required. However, due to the lack of individual data, the results could only be compared within each enrolled study; these results are presented in **Table 3**.

Publication bias, which could have had a most severe impact on the meta-analysis results, was analyzed using the asymmetry of funnel plots and Egger’s test. Fortunately, only one publication bias was found when the relationship between total hip BMD and leptin in men was pooled and analyzed. A significant correlation was found between serum leptin and total hip BMD values, analyzed by using pooled correlation. Therefore, the publication bias could be corrected through additional research.

The bone–fat interaction is quite complex, and the precise mechanism has not been elucidated (126). Osteoblasts and adipocytes that make up bone and fat, respectively, originate from the same progenitor called MSCs (7). Therefore, the relationship between bone marrow fat and bone density is inversely proportional to each other (42, 48). The ratio of bone marrow fat increases during menopause, aging, and chronic renal failure, indicating a decrease in bone density and an increase in fracture risk (127). Therefore, it is necessary to study the interaction of ghrelin, which is related to hunger or appetite, or various adipokines mainly produced in adipocytes with osteocytes, osteoblasts, and osteoclasts.

Osteoporosis is a disease in which bone quality deteriorates, and the quantity decreases, which increases the risk of fractures (5). The incidence of osteoporosis is rapidly increasing with the increase in life expectancy. Failure to prevent subsequent fractures in osteoporosis patients leads to an exponential increase in morbidity and mortality (127). Furthermore, osteoporosis has recently emerged as a serious public health concern (128). To prevent, diagnose, treat, and manage osteoporosis, biomarkers are needed. Vitamin D, osteocalcin, and procollagen type 1 N-terminal propeptide are known representative biomarkers (104). Various studies are being conducted to identify additional biomarkers or therapeutic targets, including adipokines and ghrelin (129, 130).

Resistin, a novel adipokine, is expected to serve as a biomarker for osteoporosis diagnosis or a therapeutic target (17, 30, 130). Therefore, many resistin-related studies were included in our meta-analysis. Many studies have been conducted on the effects of adipokines, especially resistin, on bone health over the past 10 years; however, no correlation was found, or insufficient data were available for meta-analysis. Nevertheless, as mentioned above, serum resistin level may have an inverse relationship with the lumbar BMD value in healthy adult men; this notion should be verified in future studies.

Studies on the correlation between visfatin level and BMD have been conducted as studies have shown that visfatin is involved in bone homeostasis and inflammation and regulates glucose metabolism associated with bone metabolism (129). However, the number of studies still needs to be increased, and there is no consistency between studies.

Our study has some limitations. Although age is a confounding factor for our analysis, we could not separate groups by detailed age due to the lack of studies. In the case of women, many studies considered menopause, so it was possible to analyze to some extent according to age roughly by dividing the group into pre and postmenopause. However, in the case of men, only some studies are separated by age. Especially, data on young men were insufficient. Although there were no significant differences in measured adipokine concentrations by adipokine source and assay approaches, their influence could not be completely ruled out. Despite these limitations, this study has several advantages. Our analysis included more studies for leptin, adiponectin, and resistin than the previous analysis. Especially, correlation studies for resistin and BMD in pre and postmenopausal women were newly added current meta-analysis. Moreover, we added data synthesis for adipokine levels in patients according to osteoporotic status. Furthermore, we confirmed publication bias in the entire group and assessed the quality of original studies. Therefore, our analysis reinforced the data quality and reliability of than previous analysis.

In conclusion, our results suggest that leptin is correlated with BMD, and adiponectin is inversely correlated with BMD. In addition, osteoporotic patients had lower leptin levels and higher adiponectin levels than the normal control. Osteoporosis patients are increasing worldwide (128). Using the serum adipokine level as an indicator, a bone density test at an appropriate time can help diagnose osteoporosis. Furthermore, an appropriate diagnosis can help improve the prognosis of many osteoporosis patients by starting treatment at the right time (131).

## Data availability statement

The original contributions presented in the study are included in the article/**Supplementary Material**. Further inquiries can be directed to the corresponding authors.

## Author contributions

SL, JeK, TG, and YK contributed to the conception and design of the study. SL and JeK conducted search, selection, and data extraction processes. YJ, JL, TG, and YK discussed the eligibility of the studies. S-KH, JaK, and KK performed the data extraction and statistical analysis. SL and JeK wrote the first draft of the manuscript. YJ, JL, KK, S-KH, JaK, TG, and YK wrote sections of the manuscript. All authors contributed to the article and approved the submitted version.
