# Supplementary material for: Effect of adipokine and ghrelin levels on BMD and fracture risk: an updated systematic review and meta-analysis
Source: Front Endocrinol (Lausanne). 2023 Apr 26;14:1044039. doi: 10.3389/fendo.2023.1044039 (PMC10171108; doi:10.3389/fendo.2023.1044039)
Supplement: Supplementary file 2 [file DataSheet_2.pdf]

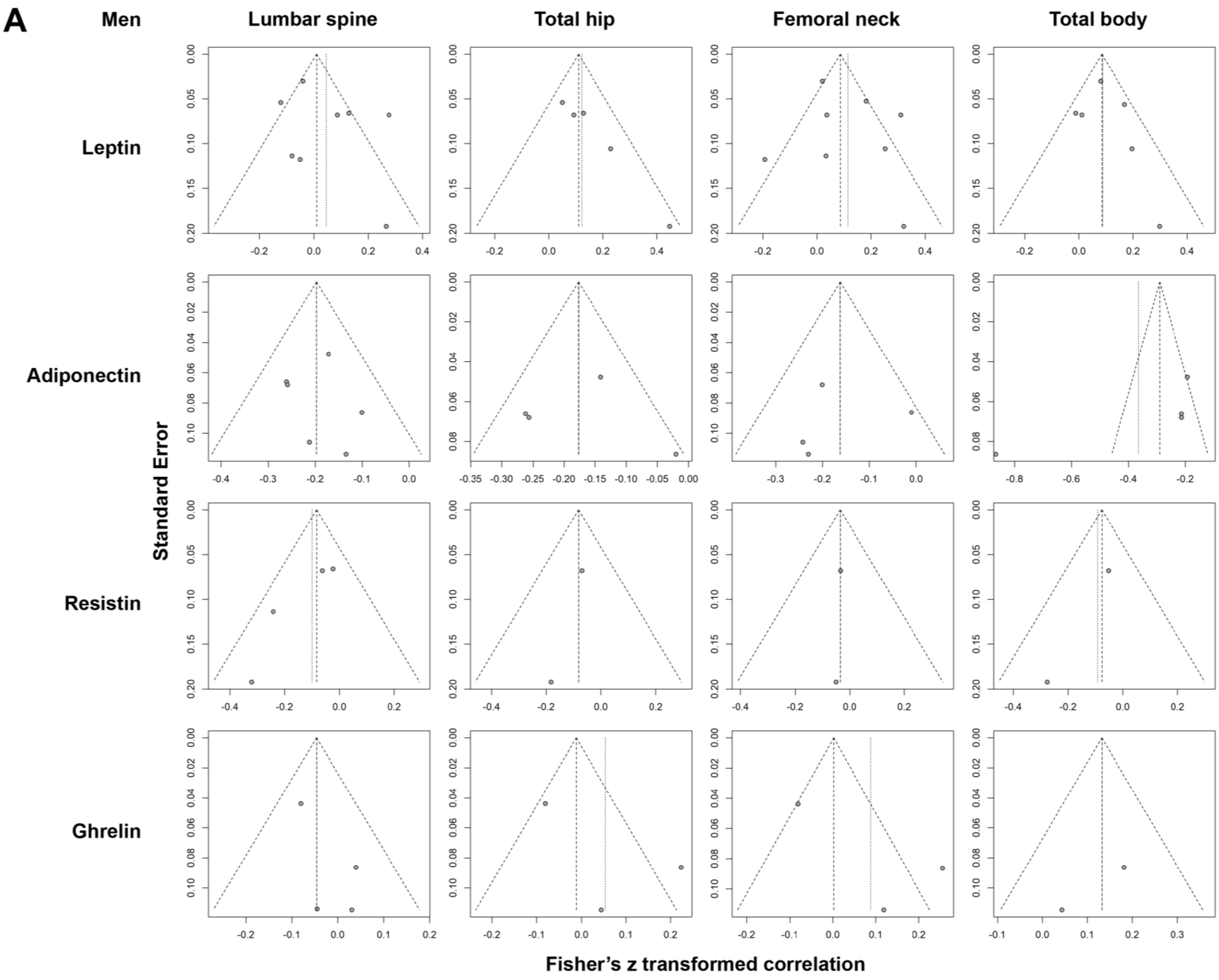
A
Men
Lumbar spine
Total hip
Femoral neck
Total body
Leptin
Adiponectin
Resistin
Ghrelin
Standard Error
Fisher's z transformed correlation

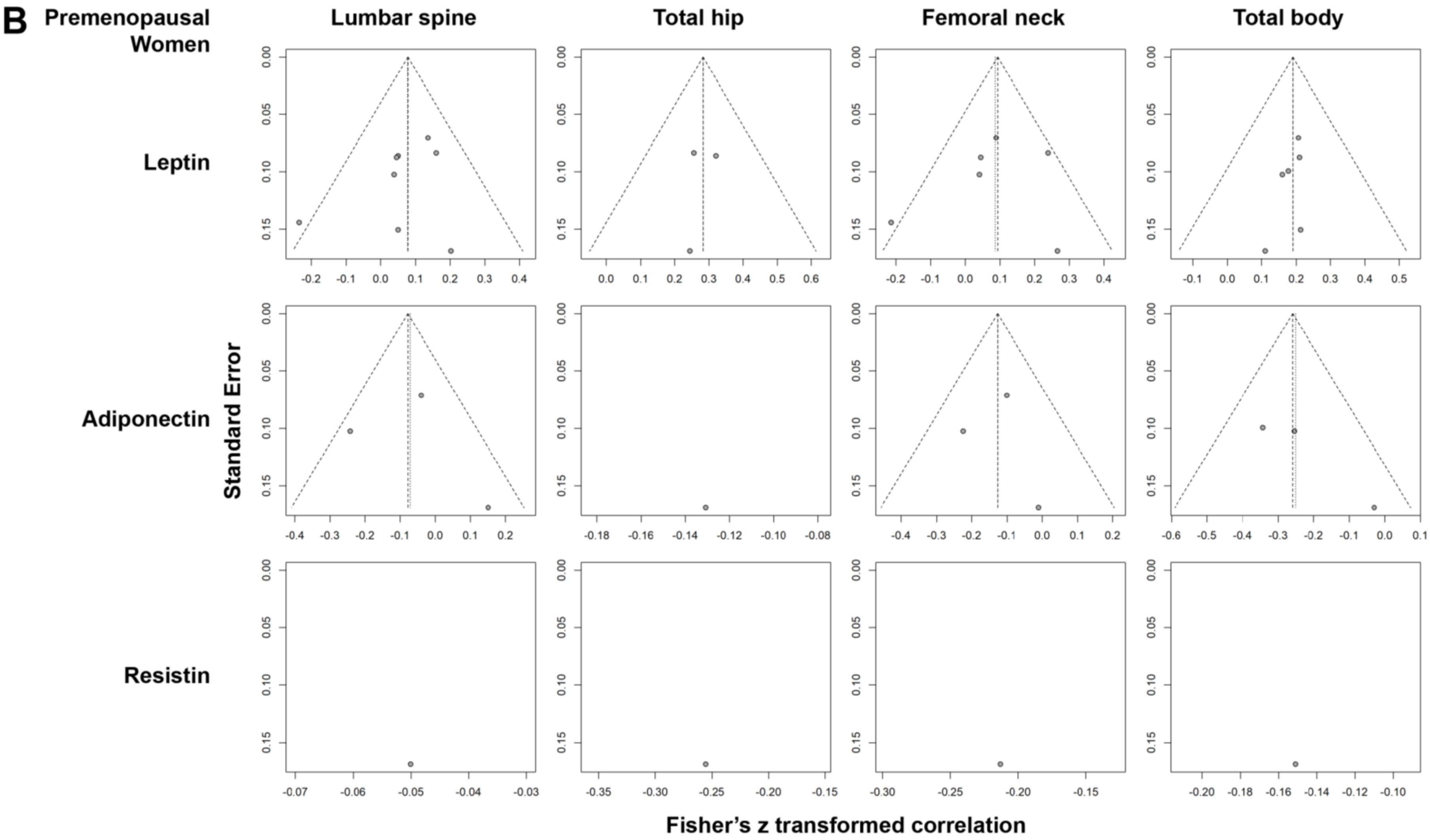
B
Premenopausal Women
Lumbar spine
Total hip
Femoral neck
Total body
Leptin
Adiponectin
Resistin
Standard Error
Fisher's z transformed correlation

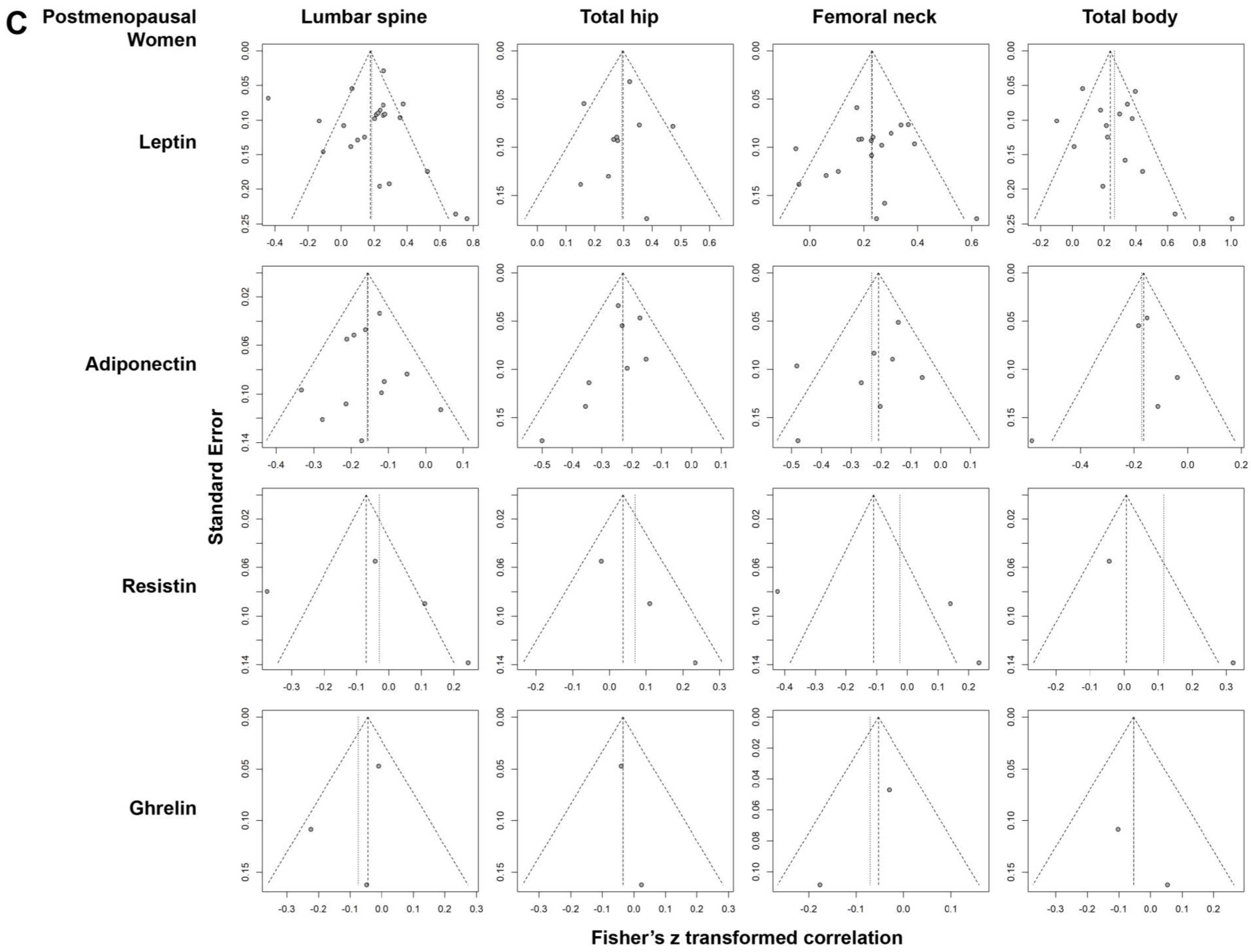
C
Postmenopausal Women
Lumbar spine
Total hip
Femoral neck
Total body
Leptin
Adiponectin
Resistin
Ghrelin
Standard Error
Fisher's z transformed correlation

Supplementary Figure S1. Funnel plots for each group

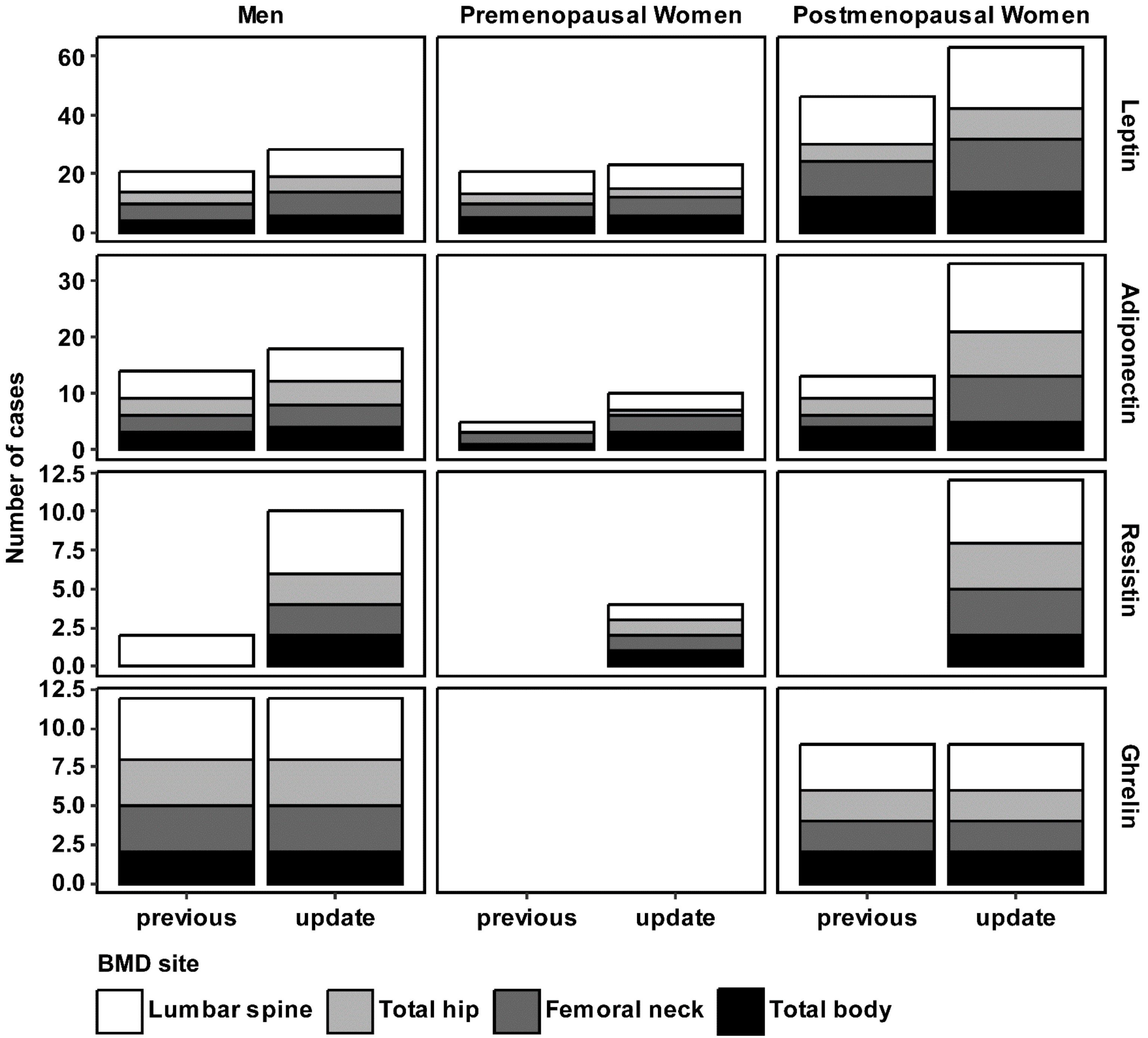

Men
Premenopausal Women
Postmenopausal Women
Leptin
Adiponectin
Resistin
Ghrelin
Number of cases
60
40
20
0
30
20
10
0
12.5
10.0
7.5
5.0
2.5
0.0
previous
update
BMD site
Lumbar spine
Total hip
Femoral neck
Total body

Supplementary Figure S2. Comparison of the number of cases to determine the correlation between adipokines or ghrelin and BMD according to sex and menopausal status

Among the studies included in the previous review, cases using the partial correlation coefficient (37,51) and cases in which menopausal status was not distinguished (64) were excluded.

previous: the number of cases in the previous review; update: the number of cases in the updated review

Supplementary Table S1. Quality assessments for cohort studies according to Newcastle-Ottawa Scale

Study (ref)	Selection				Comparability of cohorts on the basis of the design or analysis	Outcome			Total
	Representativeness of the exposed cohort	Selection of the non exposed cohort	Ascertainment of exposure	Demonstration that outcome of interest was not present at start of study		Assessment of outcome	Was follow-up long enough for outcomes to occur	Adequacy of follow up of cohorts	
Anastasilakis, 2012 (27)	1	1			1	1	1	1	6
Araneta, 2009 (29)	1	1			1	1	1	1	6
Barbour, 2011 (31)	1	1		1	2	1	1		7
Barbour, 2012 (32)	1	1		1	1	1	1		6
Breuil, 2015 (39)	1	1	1		1		1	1	6
Dennison, 2004 (46)	1	1	1	1	1	1	1	1	8
Di Carlo, 2007 (47)	1	1	1	1	1	1		1	7
Fuggle, 2018 (49)	1	1	1	1	1		1	1	7
Gulin, 2017 (52)	1	1		1	1	1	1	1	7
Johansson, 2014 (57)	1	1	1	1	1	1	1	1	8
Johansson, 2012 (58)	1	1	1	1	1	1	1	1	8
Jurimae, 2009 (63)	1	1		1	1		1	1	6
Liu, 2020 (73)	1	1	1	1	1	1	1	1	8
Nakamura, 2020 (85)	1	1	1		1	1	1	1	7
Schett, 2004 (102)	1	1	1	1	1	1	1	1	8
Shen, 2012 (105)	1	1	1	1	1		1	1	7
Tariq, 2015 (109)	1	1		1	1			1	5
Weiss, 2006 (115)	1	1		1	1		1	1	6
Weiss, 2006 (116)	1	1		1	1	1	1		6

Supplementary Table S2. Quality assessments for cross-sectional studies according to modified Newcastle-Ottawa Scale

Study (ref)	Selection				Comparability based on the study design or analysis	Outcome		Total
	Representativeness of the sample	Sample size	Non-respondents	Ascertainment of the exposure (risk factor)		Assessment of the outcome	Statistical test	
Mpalaris, 2016 (84)	1	1		2	1	2	1	8
Agbaht, 2009 (25)	1	1		2	1	2	1	8
Amini, 2013 (26)	1	1		2	2	2	1	9
Ansari, 2020 (28)	1	1	1	2	2	2	1	10
Azizieh, 2019 (30)	1	1		2	2	2	1	9
Basurto, 2009 (33)	1	1		2	2	2	1	9
Bi, 2020 (34)	1	1		2	1	2	1	8
Bilha, 2020 (35)	1	1		2	1	2	1	8
Bilha, 2018 (14)	1	1		2	2	2	1	9
Bilha, 2018 (15)	1	1		2	1	2	1	8
Blain, 2002 (36)	1	1	1	2	1	2	1	9
Blum, 2003 (37)	1	1		1	1	1	1	6
Breuil, 2011 (38)	1	1		2	1	2	1	8
Breuil, 2014 (40)	1	1		2	2	2	1	9
Canhao, 2008 (41)	1	1		2	2	2	1	9
Cervellati, 2016 (16)	1	1		2	2	2	1	9
Chan, 2018 (42)	1	1		1	1	2	1	7
Chanprasertyothin, 2005 (43)	1	1		2	1	2	1	8
Chanprasertyothin, 2006 (44)	1	1		2	1	2	1	8
Crabbe, 2006 (45)	1	1		2	2	2	1	9
Di Monaco, 2003 (48)	1	1		2	1	2	1	8
Gonnelli, 2008 (50)	1	1	1	2	1	2	1	9
Goulding, 1998 (51)	1	1		2	1	2	1	8
Haam, 2017 (53)	1	1		2	2	2	1	9
Ibrahim, 2011 (54)	1	1		2	1	2	1	8

Iwamoto, 2000 (55)	1	1		2	1	2	1	8
Jiang, 2008 (56)	1	1		2	1	2	1	8
Jurimae, 2006 (59)	1	1		2	1	2	1	8
Jurimae, 2007 (60)	1	1		2	1	2	1	8
Jurimae, 2007 (61)	1	1		2	1	2	1	8
Jurimae, 2008 (62)	1	1		2	1	2	1	8
Jurimae, 2005 (64)	1	1		2	1	2	1	8
Kim, 2014 (65)	1	1		2	1	2	1	8
Kim, 2012 (66)	1	1		2	1	2	1	8
Kim, 2008 (67)	1	1		2	2	2	1	9
King, 2010 (68)	1	1		2	1	2	1	8
Kocyigit, 2013 (69)	1	1		2	1	2	1	8
Kontogianni, 2004 (70)	1	1		2	1	2	1	8
Lee, 2014 (71)	1	1		2	1	2	1	8
Li, 2014 (72)	1	1		2	1	2	1	8
Liu, 2008 (74)	1	1		2	1	2	1	8
Liu, 2013 (75)	1	1		2	1	2	1	8
Lorentzon, 2006 (76)	1	1		2	1	2	1	8
Makovey, 2007 (77)	1	1		2	1	2	1	8
Martini, 2001 (78)	1	1		2	2	2	1	9
Michaelsson, 2008 (79)	1	1		2	1	2	1	8
Mihai, 2019 (80)	1	1		2	1	2	1	8
Mohiti-Ardekani, 2014 (81)	1	1		2	1	2	1	8
Morberg, 2003 (82)	1	1		2	1	2	1	8
Morcov, 2012 (83)	1	1		2	1	2		7
Nouh, 2012 (86)	1	1		2	1	2	1	8
Odabasi, 2000 (87)	1	1		1	1	2	1	7
Oguz, 2009 (88)	1	1		2	1	2	1	8
Oh, 2005 (89)	1	1		2	1	2	1	8
Ozkurt, 2009 (90)	1	1		2	1	2	1	8

Papadopoulou, 2004 (91)	1	1		2	1	2	1	8
Pasco, 2001 (92)	1	1		2	1	2	1	8
Pedone, 2013 (93)	1	1		2	1	2	1	8
Peng, 2008 (94)	1	1		2	1	2	1	8
Pluskiewicz, 2012 (95)	1	1		2	1	2	1	8
Rauch, 1998 (96)	1	1		2	1	2	1	8
Richards, 2007 (97)	1	1		2	1	2	1	8
Roux, 2003 (98)	1	1		2	1	2	1	8
Ruhl, 2002 (99)	1	1		2	1	2	1	8
Sahin, 2003 (100)	1	1		2	1	2	1	8
Scariano, 2003 (101)	1	1		2	1	2	1	8
Shaarawy, 2003 (103)	1	1		2	1	2	1	8
Shabat, 2009 (104)	1	1		2	1	2	1	8
Sherk, 2011 (106)	1	1		2	1	2	1	8
Sun, 2003 (107)	1	1		2	1	2	1	8
Tanna, 2017 (108)	1	1		2	1	2	1	8
Tariq, 2021 (17)	1	1		2	1	2	1	8
Tenta, 2012 (110)	1	1		2	1	2	1	8
Thomas, 2001 (111)	1	1		2	1	2	1	8
Tohidi, 2012 (112)	1	1		2	1	2	1	8
Ushiroyama, 2003 (113)	1	1		2	1	2	1	8
Varri, 2016 (114)	1	1		2	1	2	1	8
Wu, 2010 (117)	1	1		2	1	2	1	8
Yamauchi, 2001 (118)	1	1		2	1	2	1	8
Yilmazi, 2005 (119)	1	1		2	1	2	1	8
Zhang, 2010 (120)	1	1		2	1	2	1	8
Zhao, 2012 (121)	1	1		2	1	2	1	8
Zhong, 2005 (122)	1	1		2	1	2	1	8
Zoico, 2003 (123)	1	1		2	1	2	1	8
Zoico, 2008 (124)	1	1		2	1	2	1	8

Supplementary Table S3. GRADE evidence profile for the correlation studies of adipokines or ghrelin with BMD

Quality assessment							Grade
No of studies	Study design	Risk of bias	Inconsistency	Indirectness	Imprecision	Other considerations	
Men, Leptin and Lumbar spine BMD							
8	observational studies	not serious	very serious[b]	not serious	not serious	none	⊕○○○ Very low
Men, Leptin and Total hip BMD							
5	observational studies	not serious	not serious	not serious	not serious	Publication bias strongly suspected[d]	⊕○○○ Very low
Men, Leptin and Femoral neck BMD							
8	observational studies	not serious	very serious[b]	not serious	not serious	none	⊕○○○ Very low
Men, Leptin and Total body BMD							
6	observational studies	not serious	not serious	not serious	not serious	none	⊕⊕○○ Low
Men, Adiponectin and Lumbar spine BMD							
6	observational studies	not serious	not serious	not serious	not serious	none	⊕⊕○○ Low
Men, Adiponectin and Total hip BMD							
4	observational studies	not serious	serious[c]	not serious	not serious	none	⊕○○○ Very low
Men, Adiponectin and Femoral neck BMD							
4	observational studies	not serious	not serious	not serious	not serious	none	⊕⊕○○ Low
Men, Adiponectin and Total body BMD							
4	observational studies	not serious	very serious[b]	not serious	not serious	none	⊕○○○ Very low
Men, Resistin and Lumbar spine BMD							
4	observational studies	not serious	not serious	not serious	not serious	none	⊕⊕○○ Low
Men, Resistin and Total hip BMD							
2	observational studies	not serious	not serious	not serious	not serious	none	⊕⊕○○ Low
Men, Resistin and Femoral neck BMD							
2	observational studies	not serious	not serious	not serious	not serious	none	⊕⊕○○ Low
Men, Resistin and Total body BMD							

2	observational studies	not serious	not serious	not serious	not serious	none	⊕⊕○○ Low
Men, Ghrelin and Lumbar spine BMD							
4	observational studies	serious[a]	not serious	not serious	not serious	none	⊕○○○ Very low
Men, Ghrelin and Total hip BMD							
3	observational studies	serious[a]	very serious[b]	not serious	not serious	none	⊕○○○ Very low
Men, Ghrelin and Femoral neck BMD							
3	observational studies	serious[a]	very serious[b]	not serious	not serious	none	⊕○○○ Very low
Men, Ghrelin and Total body BMD							
2	observational studies	not serious	not serious	not serious	not serious	none	⊕⊕○○ Low
Premenopausal women, Leptin and Lumbar spine BMD							
8	observational studies	not serious	not serious	not serious	not serious	none	⊕⊕○○ Low
Premenopausal women, Leptin and Total hip BMD							
3	observational studies	not serious	not serious	not serious	not serious	none	⊕⊕○○ Low
Premenopausal women, Leptin and Femoral neck BMD							
6	observational studies	not serious	not serious	not serious	not serious	none	⊕⊕○○ Low
Premenopausal women, Leptin and Total body BMD							
6	observational studies	not serious	not serious	not serious	not serious	none	⊕⊕○○ Low
Premenopausal women, Adiponectin and Lumbar spine BMD							
3	observational studies	not serious	serious[c]	not serious	not serious	none	⊕○○○ Very low
Premenopausal women, Adiponectin and Total hip BMD							
1	observational study	not serious	NA	not serious	not serious	none	NA[e]
Premenopausal women, Adiponectin and Femoral neck BMD							
3	observational studies	not serious	not serious	not serious	not serious	none	⊕⊕○○ Low
Premenopausal women, Adiponectin and Total body BMD							
3	observational studies	not serious	not serious	not serious	not serious	none	⊕⊕○○ Low
Premenopausal women, Resistin and Lumbar spine BMD							

1	observational study	not serious	NA	not serious	not serious	none	NA[e]
Premenopausal women, Resistin and Total hip BMD							
1	observational study	not serious	NA	not serious	not serious	none	NA[e]
Premenopausal women, Resistin and Femoral neck BMD							
1	observational study	not serious	NA	not serious	not serious	none	NA[e]
Premenopausal women, Resistin and Total body BMD							
1	observational study	not serious	NA	not serious	not serious	none	NA[e]
Postmenopausal women, Leptin and Lumbar spine BMD							
21	observational studies	serious[a]	very serious[b]	not serious	not serious	none	⊕○○○ Very low
Postmenopausal women, Leptin and Total hip BMD							
10	observational studies	not serious	not serious	not serious	not serious	none	⊕⊕○○ Low
Postmenopausal women, Leptin and Femoral neck BMD							
18	observational studies	not serious	not serious	not serious	not serious	none	⊕⊕○○ Low
Postmenopausal women, Leptin and Total body BMD							
14	observational studies	not serious	very serious[b]	not serious	not serious	none	⊕○○○ Very low
Postmenopausal women, Adiponectin and Lumbar spine BMD							
12	observational studies	not serious	not serious	not serious	not serious	none	⊕⊕○○ Low
Postmenopausal women, Adiponectin and Total hip BMD							
8	observational studies	not serious	not serious	not serious	not serious	none	⊕⊕○○ Low
Postmenopausal women, Adiponectin and Femoral neck BMD							
8	observational studies	not serious	serious[c]	not serious	not serious	none	⊕○○○ Very low
Postmenopausal women, Adiponectin and Total body BMD							
5	observational studies	not serious	not serious	not serious	not serious	none	⊕⊕○○ Low
Postmenopausal women, Resistin and Lumbar spine BMD							
4	observational studies	not serious	very serious[b]	not serious	not serious	none	⊕○○○ Very low
Postmenopausal women, Resistin and Total hip BMD							
3	observational studies	not serious	not serious	not serious	not serious	none	⊕⊕○○ Low

Postmenopausal women, Resistin and Femoral neck BMD							
3	observational studies	not serious	very serious[b]	not serious	not serious	none	⊕◯◯◯ Very low
Postmenopausal women, Resistin and Total body BMD							
2	observational studies	not serious	very serious[b]	not serious	not serious	none	⊕◯◯◯ Very low
Postmenopausal women, Ghrelin and Lumbar spine BMD							
3	observational studies	serious[a]	not serious	not serious	not serious	none	⊕◯◯◯ Very low
Postmenopausal women, Ghrelin and Total hip BMD							
2	observational studies	serious[a]	not serious	not serious	not serious	none	⊕◯◯◯ Very low
Postmenopausal women, Ghrelin and Femoral neck BMD							
2	observational studies	serious[a]	not serious	not serious	not serious	none	⊕◯◯◯ Very low
Postmenopausal women, Ghrelin and Total body BMD							
2	observational studies	not serious	not serious	not serious	not serious	none	⊕⊕◯◯ Low

[a] Studies with a NOS total score of less than 7(cohort) or 8(cross-sectional) were included.

[b] Considerable heterogeneity ($I^2 > 75\%$).

[c] Substantial heterogeneity ($50\% < I^2 <= 75\%$)

[d] According to the Egger's test

[e] Heterogeneity and publication bias could not assess due to only one study included.

Supplementary Table S4. Subgroup analysis for different geographical populations of men in Table 1

Adipokine/ghrelin	BMD site	Region		No. of patients	Heterogeneity		Random effects model	
					I^2	p	r	p
Leptin	Lumbar spine	Total		2266	78	< 0.001	0.05	0.38
		Asia	China (72, 94), Thailand (43), Korea (89)	606	17	0.30	0.06	0.20
		Europe	Romania (14), Sweden (76), UK (46)	1317	90	< 0.001	0.15	0.28
		North America	USA (111)	343	NA	NA	-0.12	0.03
	Total hip	Total		916	30	0.22	0.12	0.004
		Asia	China (72, 94)	451	0	0.71	0.11	0.02
		Europe	Italy (123), Romania (14)	122	0	0.32	0.27	0.003
		North America	USA (111)	343	NA	NA	0.05	0.37
	Femoral neck	Total		2146	76	< 0.001	0.11	0.03
		Asia	China (72), Korea (89), Thailand (43)	374	33	0.22	-0.02	0.75
		Europe	Greece (91), Italy (123), Romania (14), Sweden (76), UK (46)	1772	82	< 0.001	0.19	0.007
	Total body	Total		1958	36	0.16	0.09	0.009
		Asia	China (72, 94)	451	0	0.82	-0.0003	0.99
		Europe	Denmark (82), Italy (123), Romania (14), Sweden (76)	1507	18	0.30	0.12	< 0.001
Adiponectin	Lumbar spine	Total		1201	0	0.61	-0.19	< 0.001
		Asia	China (72, 94), Korea (89)	531	0	0.59	-0.24	< 0.001
		Europe	Italy (50), Sweden (79)	578	0	0.47	-0.15	< 0.001
		North America	Mexico (33)	92	NA	NA	-0.21	0.045
	Total hip	Total		1029	56	0.08	-0.17	< 0.001
		Asia	China (72, 94)	451	0	0.96	-0.25	< 0.001
		Europe	Italy (50), Sweden (79)	578	33	0.22	-0.10	0.07
	Femoral neck	Total		528	31	0.23	-0.16	0.003
		Asia	China (72), Korea (89)	299	0	0.83	-0.21	< 0.001
		Europe	Italy (50)	137	NA	NA	-0.01	0.91
		North America	Mexico (33)	92	NA	NA	-0.24	0.02
	Total body	Total		1029	94	< 0.001	-0.35	0.007
		Asia	China (72, 94)	451	0	1.00	-0.21	< 0.001
		Europe	Italy (50), Sweden (79)	578	98	< 0.001	-0.48	0.12
Resistin	Lumbar spine	Total		561	31	0.22	-0.1	0.07
		Asia	China (72, 94), Korea (89)	531	28	0.25	-0.08	0.14
		Europe	Romania (14)	30	NA	NA	-0.31	0.10
	Total hip	Total		249	0	0.58	-0.08	0.2
		Asia	China (72)	219	NA	NA	-0.07	0.30
		Europe	Romania (14)	30	NA	NA	-0.18	0.34
	Femoral neck	Total		249	0	0.93	-0.03	0.59
		Asia	China (72)	219	NA	NA	-0.03	0.63
		Europe	Romania (14)	30	NA	NA	-0.05	0.79
	Total body	Total		249	18	0.27	-0.09	0.28
		Asia	China (72)	219	NA	NA	-0.05	0.44
		Europe	Romania (14)	30	NA	NA	-0.27	0.15
Ghrelin	Lumbar spine	Total		821	0	0.56	-0.05	0.19
		Asia	Korea (89)	80	NA	NA	-0.05	0.69
		Europe	Italy (50)	137	NA	NA	0.04	0.64
		North America	USA (116)	525	NA	NA	-0.08	0.07
		Oceania	Australia (77)	79	NA	NA	0.03	0.79
	Total hip	Total		741	80	0.006	0.05	0.6
		Europe	Italy (50)	137	NA	NA	0.22	0.01
		North America	USA (116)	525	NA	NA	-0.08	0.07
		Oceania	Australia (77)	79	NA	NA	0.05	0.69
	Femoral neck	Total		742	85	0.001	0.09	0.45

		Asia	Korea (89)	80	NA	NA	0.12	0.23
		Europe	Italy (50)	137	NA	NA	0.25	0.003
		North America	USA (116)	525	NA	NA	-0.08	0.07
		Total		216	0	0.34	0.13	0.06
	Total body	Europe	Italy (50)	137	NA	NA	0.18	0.04
		Oceania	Australia (77)	79	NA	NA	0.04	0.70

NA: not applicable

Supplementary Table S5. Subgroup analysis for different geographical populations of premenopausal women in Table 1

Adipokine/ ghrelin	BMD site	Region		No. of patients	Heterogeneity		Random effects model	
					I^2	p	r	p
Leptin	Lumbar spine	Total		853	4	0.4	0.08	0.03
		Asia	Japan (55), Korea (67), Thailand (43)	243	64	0.06	0.01	0.92
		Europe	Estonia (59, 60), Romania (15)	340	0	0.63	0.12	0.04
		North America	USA (111)	137	NA	NA	0.05	0.56
		Oceania	Australia (92)	133	NA	NA	0.05	0.60
	Total hip	Total		320	0	0.84	0.28	< 0.001
		Asia	Korea (67)	145	NA	NA	0.25	0.002
		Europe	Romania (15)	38	NA	NA	0.24	0.15
		North America	USA (111)	137	NA	NA	0.31	< 0.001
	Femoral neck	Total		669	45	0.1	0.09	0.12
		Asia	Korea (67), Thailand (43)	196	86	0.007	0.03	0.90
		Europe	Estonia (59, 60), Romania (15)	340	0	0.52	0.09	0.09
		Oceania	Australia (92)	133	NA	NA	0.05	0.61
	Total body	Total		624	0	0.99	0.19	< 0.001
		Asia	Japan (55)	47	NA	NA	0.21	0.16
		Europe	Estonia (59, 60), Romania (15)	340	0	0.84	0.18	< 0.001
		North America	USA (68)	104	NA	NA	0.18	0.07
		Oceania	Australia (92)	133	NA	NA	0.21	0.02
Adiponectin	Lumbar spine	Total		336	57	0.1	-0.07	0.45
		Asia	Thailand (44)	200	NA	NA	-0.04	0.57
		Europe	Estonia (60), Romania (15)	136	75	0.046	-0.07	0.73
	Total hip	Europe	Romania (15)	38	NA	NA	-0.13	0.44
	Femoral neck	Total		336	0	0.47	-0.13	0.02
		Asia	Thailand (44)	200	NA	NA	-0.10	0.16
		Europe	Estonia (60), Romania (15)	136	14	0.28	-0.16	0.11
	Total body	Total		240	22	0.28	-0.25	< 0.001
		Europe	Estonia (60), Romania (15)	136	22	0.26	-0.18	0.08
		North America	USA (68)	104	NA	NA	-0.33	< 0.001
Resistin	Lumbar spine	Europe	Romania (15)	38	NA	NA	-0.05	0.77
	Total hip	Europe	Romania (15)	38	NA	NA	-0.25	0.13
	Femoral neck	Europe	Romania (15)	38	NA	NA	-0.21	0.21
	Total body	Europe	Romania (15)	38	NA	NA	-0.15	0.37

NA: not applicable

Supplementary Table S6. Subgroup analysis for different geographical populations of postmenopausal women in Table 1

Adipokine/ ghrelin	BMD site		Region	No. of patients	Heterogeneity		Random effects model	
					I^2	p	r	p
Leptin	Lumbar spine	Total		3456	84	< 0.001	0.18	< 0.001
		Asia	China (120), Japan (55, 85, 113, 118), Korea (67), Thailand (43), Turkey (87, 88, 100)	2367	91	< 0.001	0.09	0.28
		Europe	Estonia (62), France (36, 38, 98), Greece (84), Italy (16, 47), Romania (15), UK (46)	857	57	0.01	0.28	< 0.001
		North America	USA (111)	165	NA	NA	0.25	0.001
		Oceania	Australia (92)	67	NA	NA	0.14	0.26
	Total hip	Total		2159	35	0.13	0.29	< 0.001
		Asia	China (120), Japan (85), Korea (67)	1422	68	0.045	0.25	< 0.001
		Europe	France (98), Italy (16, 48, 123, 124), Romania (15)	572	0	0.82	0.28	< 0.001
		North America	USA (111)	165	NA	NA	0.44	< 0.001
	Femoral neck	Total		1965	42	0.03	0.22	< 0.001
		Asia	Japan (118), Korea (67), Thailand (43), Turkey (88, 100)	542	52	0.08	0.16	0.01
		Europe	Estonia (62), Finland (114), France (36, 38, 98), Greece (84), Italy (16, 123, 124), Romania (15), UK (46)	1313	40	0.08	0.26	< 0.001
		North America	USA (106)	43	NA	NA	0.27	0.08
		Oceania	Australia (92)	67	NA	NA	0.11	0.39
	Total body	Total		1625	72	< 0.001	0.26	< 0.001
		Asia	China (120), Japan (55, 118), Turkey (100)	604	37	0.19	0.07	0.23
		Europe	Estonia (62), Finland (114), France (36), Italy (47, 78, 123, 124), Romania (15)	911	55	0.02	0.34	< 0.001
		North America	USA (106)	43	NA	NA	0.32	0.04
		Oceania	Australia (92)	67	NA	NA	0.22	0.08
Adiponectin	Lumbar spine	Total		2850	9	0.36	-0.16	< 0.001
		Asia	China (120), Iran (112), Japan (85), Korea (66), Kuwait (30), Turkey (90)	1932	3	0.40	-0.15	< 0.001
		Europe	Estonia (62), Greece (84, 110), Italy (16), Romania (15), Sweden (79)	918	28	0.23	-0.16	< 0.001
	Total hip	Total		2053	0	0.46	-0.23	< 0.001
		Asia	China (120), Japan (85), Turkey (90)	1298	0	0.95	-0.23	< 0.001
		Europe	Italy (16, 124), Poland (95), Romania (15), Sweden (79)	755	37	0.18	-0.24	< 0.001
	Femoral neck	Total		1024	52	0.04	-0.23	< 0.001
		Asia	Iran (112), Korea (66)	528	0	0.40	-0.16	< 0.001
		Europe	Estonia (62), Greece (84), Italy (16, 124), Poland (95), Romania (15)	496	57	0.04	-0.26	< 0.001
	Total body	Total		972	46	0.12	-0.17	0.001
		Asia	China (120)	336	NA	NA	-0.18	< 0.001
		Europe	Estonia (62), Italy (124), Romania (15), Sweden (79)	636	59	0.06	-0.18	0.03
Resistin	Lumbar spine	Total		678	88	< 0.001	-0.03	0.8
		Asia	China (120), Pakistan (17)	496	92	< 0.001	-0.20	0.22
		Europe	Italy (16), Romania (15)	182	0	0.42	0.15	0.046
	Total hip	Total		518	48	0.15	0.07	0.33
		Asia	China (120)	336	NA	NA	-0.02	0.69
		Europe	Italy (16), Romania (15)	182	0	0.45	0.15	0.051
	Femoral neck	Total		342	93	< 0.001	-0.02	0.91
		Asia	Pakistan (17)	160	NA	NA	-0.40	< 0.001
		Europe	Italy (16), Romania (15)	182	0	0.57	0.17	0.03
	Total body	Total		391	83	0.01	0.12	0.52
		Asia	China (120)	336	NA	NA	-0.04	0.43
		Europe	Romania (15)	55	NA	NA	0.31	0.02
Ghrelin	Lumbar spine	Total		581	39	0.19	-0.07	0.29
		Europe	Estonia (62)	88	NA	NA	-0.22	0.04
		North America	USA (116)	452	NA	NA	-0.01	0.83
		Oceania	Australia (77)	41	NA	NA	-0.05	0.77
	Total hip	Total		493	0	0.7	-0.04	0.44
		North America	USA (116)	452	NA	NA	-0.04	0.40

		Oceania	Australia (77)	41	NA	NA	0.03	0.88
	Femoral neck	Total		540	35	0.22	-0.07	0.28
		Europe	Estonia (62)	88	NA	NA	-0.1750	0.10
		North America	USA (116)	452	NA	NA	-0.0300	0.52
	Total body	Total		129	0	0.42	-0.05	0.55
		Europe	Estonia (62)	88	NA	NA	-0.10	0.35
		Oceania	Australia (77)	41	NA	NA	0.05	0.74

NA: not applicable

Supplementary Table S7. Results of multiple regression analysis of 16 studies that assessed the association between BMD and adiponectin levels based on sex and menopausal status (16, 28, 33, 60, 62, 64, 66, 68, 70, 72, 94, 110, 112, 117, 120, 124)

Group	References	Number of patients	Age (m ± SD and/or min - max)	BMD (dependent variable)	Independent variables	R^2	Significant independent variables	Regression coefficient β	β p-value
Men	Basurto 2009	92	64.3 ± 7.7	Lumbar spine	age, BMI, adiponectin	0.071	NA	-0.074	0.514
				Femoral Neck		0.090	BMI	-0.016	0.887
	Li 2014	219	46.37 ± 15.67	Lumbar spine	LM, adiponectin	NA	LM, adiponectin	-0.163	NA
				Total hip	LM, adiponectin, age		LM, adiponectin, age	-0.137	
				Total body	LM, FM, adiponectin		LM, FM, adiponectin	-0.187	
	Peng 2008	232	20–80	Total body	age, BMI, FM, LM, smoking habits, adiponectin, leptin, resistin, visfatin	0.222	LM, FM, adiponectin	-0.178	**0.012**
				Lumbar spine		0.233	LM, adiponectin	−0.163	**0.025**
				Total hip		0.206	LM, adiponectin, age	−0.148	**0.046**
Pre-menopausal women	Jurimae 2005	38 Pre (21) and postmenopausal (17) women	45.1 ± 4.2	Total body	FM, adiponectin, menopausal status	0.442	NA	-0.152	**0.002**
			53.5 ± 5.7	Lumbar spine		0.295		-0.283	**0.006**
	Jurimae 2007	98 Sedentary	45.2 ± 4.3	Total body	BMI, FM, trunk fat, trunk fat/leg fat ratio, FFM, adiponectin, leptin, IGF1, Insulin, fasting insulin resistance index.	0.4	FM, FFM, adiponectin	-0.01	**0.018**
				Femoral neck		0.39	FFM, adiponectin, FM	-0.01	**0.002**
				Lumbar spine		0.3	FFM, trunk fat/leg fat ratio, adiponectin	-0.01	**0.043**
	King 2010	104	43.1 ± 0.4	Total body	leptin, adiponectin, BMI, percentage body fat, FM, LM, body mass	0.07	adiponectin	-0.26	**0.004**
	Kontogianni 2004	80 Perimenopausal women	42 - 68	Lumbar spine	age, menopausal status, smoking habits, calcium intake, physical activity index, BMI, BF%, LM, insulin, cortisol, T, FSH, leptin, adiponectin.	0.361	age, BMI, insulin, leptin	NA	NA
	Wu 2010	265	34.6 ± 7.6	Total Body	Age, YSM, BMI, FM, LM, smoking habits, calcium intake, physical activity, 25OH D, PTH, E2, T, insulin, adiponectin, leptin	0.243	Age, E2, LM	NA	NA
				Lumbar spine		0.187	Age, E2		
				Total Hip		0.223	Age, E2		
				Total Forearm		0.201	Age, E2, LM		
Post-menopausal women	Ansari 2020	175: control(55), low BMD(120)	control(52.4±7.9), low BMD(56.2±8.3)	Femoral neck	adiponectin, resistin, lipocalin-2, adipsin, leptin, insulin, RankL, 25OHD	NA	adiponectin	-0.27	**0.002**
	Cervellati 2016	127: normal(31), osteopenia(53), osteoporosis(43)	normal(55.3±0.7), osteopenia(56.7±0.6), osteoporosis(58.5±0.4)	Trochanter	adiponectin, age, waist circumference	NA	age, waist circumference	-0.05	NA
	Jurimae 2008	88	68.9 ± 6.8 58 - 80	Total body	BMI, WHR, WTR, FM, FFM, ghrelin, adiponectin, leptin, IGF-I, insulin, HOMA	0.35	FM, FFM, leptin	NA	NA
				Lumbar spine		0.3	FM, adiponectin, ghrelin	-0.006	**0.04**
				Femoral neck		0.22	FM, FFM, leptin	NA	NA
	Kim 2012	146: normal(56), osteopenia(54), osteoporosis(36)	normal(56.63±6.25), osteopenia(57.48±5.55), osteoporosis(58.67±6.40)	Femoral neck	mitochondrial DNA copy number, adiponectin, osteocalcin, HDL-cholesterol	0.26	mitochondrial DNA copy number, adiponectin, osteocalcin	-0.047	**<0.001**
	Tenta 2012	81: normal(38), osteopenic/ osteoporotic(43)	normal(53.7±4.5), osteopenic/ osteoporotic(55.5±4.2)	Lumbar spine	age, LM, smoking, OPG/sRANKL, IGF-1, adiponectin	0.079	LM	0.126	0.3
	Tohidi 2012	382	59 ± 7.5	Lumbar spine	age, BMI, C-reactive protein, osteoprotegrein, RANKL,	NA	omentin-1	-0.06	0.248

				Femoral neck	CrossLaps, osteocalcin, alkaline phosphatase, smoking, hormone replacement therapy, calcium, vitamin D, omentin-1, visfatin, adiponectin		NA	-0.02	0.725
	Wu 2010	336	56.9 ± 6.5	Total Body	Age, YSM, BMI, FM, LM, smoking habits, calcium intake, physical activity, 25OHD, PTH, E2, T, insulin, adiponectin, leptin	0.198	YSM, LM, adiponectin, E2	-0.105	**0.043**
				Lumbar spine		0.214	YSM, LM, adiponectin, E2, BMI	-0.103	**0.048**
				Total Hip		0.241	YSM, LM, adiponectin, E2	-0.112	**0.028**
				Total Forearm		0.244	YSM, LM, adiponectin	-0.125	**0.014**
	Zhang 2010	336	56.9 ± 6.5	Total body	YSM, LM, adiponectin, E2	0.236	YSM, LM, adiponectin, E2	-2.54*	**0.012**
				Lumbar spine	YSM, adiponectin, E2, BMI	0.256	YSM, adiponectin, E2, BMI	-2.684*	**0.008**
				Total hip	YSM, LM, adiponectin, E2, trunk fat:leg fat ratio	0.273	YSM, LM, adiponectin, E2, trunk fat:leg fat ratio	-2.247*	**0.025**
				Total forearm	YSM, LM, adiponectin, E2	0.263	YSM, LM, adiponectin, E2	-2.167*	**0.034**
	Zoico 2008	36	70.8 ± 2.6<br>66 - 77	Total body	BMI, FM, leptin, adiponectin, HOMA, DHEAS	0.387	FM, adiponectin	-0.385	**0.01**
				Femoral neck		0.174	adiponectin	-0.445	**0.007**

*unstandardized coefficients

25OHD, 25 hydroxyvitamin D; BMI, body mass index; DHEA, dehydroepiandrosterone sulfate; E2, estradiol; FFM, fat free mass; FM, fat mass; HOMA, homeostasis model assessment; LM, lean mass; NA, not applicable; PTH, parathormone; T, testosterone; WHR, waist-to-hip ratio; WTR, waist-to-thigh ratio; YSM, ears since menopause.

Supplementary Table S8. Results of multiple regression analysis of 25 studies that assessed the association between BMD and leptin levels based on sex and menopausal status (16, 28, 35-37, 43, 45, 48, 59, 60, 62, 70, 76, 82, 89, 92, 94, 99, 107, 114, 117, 118, 122-124)

Group	References	Number of patients	Age (m ± SD and/or min - max)	BMD (dependent variable)	Independent variables	R^2	Significant independent variables	Regression coefficient β	β p-value
Men	Chanprasertyothin 2005	75	52 ± 15.8	Lumbar spine	Age, BMI, leptin, T, E2	NA	Age, BMI, leptin, T, E2	-0.36	**< 0.05**
				Femoral neck			Age, BMI, leptin, E2	-0.32	**< 0.05**
	Crabbe 2006	270	71 - 86	Forearm	Age, FM, E2, leptin, leptin receptor genotype	NA	Age, FM, E2	NA	0.21
				Total Hip			Age, FM, E2		0.18
	Lorentzon 2006	1068 between 18 and 20 yr	18.9 ± 0.6	Total body	Total body adipose tissue and LM, leptin, age, height, present physical activity (h/wk), calcium intake, smoking.	0.444	NA	-0.08	**0.01**
				Lumbar spine		0.245		-0.13	**< 0.01**
				Femoral neck		0.304		-0.06	0.09
				Trochanter		0.303		-0.09	**0.01**
				Radius dominant		0.253		-0.07	0.07
				Radius nondominant		0.241		-0.06	0.11
	Morberg 2003	323	49.9 ± 6	Total body	Leptin, FM, LM, age, smoking, Occupational physical activity, Nonoccupational physical activity	NA	FM, LM, occupational physical activity,	-0.002	NA
	Oh 2005	80 middle-aged	54.5 ± 6.4	lumbar spine	Age, BMI, log(leptin), Resistin, Triglyceride, E2, T	0.256	Age, BMI, resistin	-0.144	0.594
	Peng 2008	232	20–80	Total body	age, BMI, FM, LM, smoking habits, adiponectin, leptin, resistin, visfatin	0.222	LM, FM, adiponectin	NA	NA
				Lumbar spine		0.233	LM, adiponectin		
				Total hip		0.206	LM, adiponectin, age		
	Ruhl 2002	2761	43 ± 0.6	Total hip	age, ethnicity, BMI, education, smoking, drinking, physical activity, dietary protein intake, thyroxine, leptin	NA	NA	0.097	**< 0.001**
	Sun 2003	50	34 ± 13	Total Body	age, BMI, E2, T, and leptin	0.631	Age, BMI, leptin	-0.01	**0.0039**
	Zoico 2003	92	71.7 ± 2.1	Total Body	Age, leptin, FM, FFM	0.036	Age	NA	NA
					Age, leptin, FM, FFM, BMI	0.094	Age, BMI		
Pre-menopausal women	Blum 2003	153	41.6 ± 0.8	Total Body	Leptin adjusted for height	0.10	Leptin	0.03	**< 0.05**
				Lumbar spine		0.05	NA	0.02	NA
				Total Hip		0.12	Leptin	0.06	**< 0.05**
	Chanprasertyothin 2005	51	36.7 ± 8.6	Lumbar spine	Age, BMI, leptin	NA	Age, BMI, leptin	-0.29	**< 0.01**
				Femoral neck				-0.29	**< 0.05**
	Jurimae 2006	204	35 ± 7.7<br>18 - 49	Total body	Age, leptin	NA	Age, leptin	0.165	**< 0.005**
				Lumbar spine				NA	**< 0.05**
				Femoral neck				NA	**< 0.05**
	Jurimae 2007	98 Sedentary	45.2 ± 4.3	Total body	BMI, FM, trunk fat, trunk fat/leg fat ratio, FFM, adiponectin, leptin, IGF1, insulin and fasting insulin resistance index.	0.4	FM, FFM, adiponectin	NA	NA
				Femoral neck		0.39	FFM, adiponectin, FM		
				Lumbar spine		0.3	FFM, trunk fat/leg fat ratio, adiponectin		
	Kontogianni 2004	80 Perimenopausal women	42 - 68	Lumbar spine	age, menopausal status, smoking habits, calcium intake, physical activity index, BMI, %Fat, LM, insulin, cortisol, T, FSH, leptin, adiponectin.	0.361	age, BMI, insulin, leptin	-0.005	**0.027**
	Pasco 2001	214: pre (133), postmenopausal (67), indeterminate (14)	20 - 91	Femoral neck	Age, weight, FM, leptin	NA	Age, weight, FM	0.034	0.115
				Ward's triangle				0.046	0.058
				Trochanter				0.031	0.12
				Lumbar spine				0.029	0.253
				Total body				0.015	0.216
	Ruhl 2002	1906	36 ± 0.3	Total hip	age, ethnicity, BMI, physical activity, BMI change, caffeine	NA	NA	-0.009	0.66

					intake, calcium intake from diet and supplements, TSH, leptin				
	Wu 2010	265	34.6 ± 7.6	Total Body	Age, YSM, BMI, FM, LM, smoking habits, calcium intake, physical activity, 25OHD, PTH, E2, T, insulin, adiponectin, leptin	0.243	Age, E2, LM	NA	NA
				Lumbar spine		0.187	Age, E2		
				Total Hip		0.223	Age, E2		
				Total Forearm		0.201	Age, E2, LM		
	Zhong 2005	340	NA	Lumbar spine	age, weight, BMI, leptin	0.005	NA	NA	NA
				Femoral Neck		0.032			
				Total hip		0.042			
				Left 1/3 radius+ulna		0.038			
Post-menopausal women	Ansari 2020	175: control(55), low BMD(120)	control(52.4±7.9), low BMD(56.2±8.3)	Femoral neck	adiponectin, resistin, lipocalin-2, adipsin, leptin, insulin, RankL, 25OHD	NA	adiponectin	0.02	0.45
	Bilha 2020	55	61 ± 1.1	Femoral neck	PTH, 25(OH)D, FGF23, Leptin	NA	FGF23	NA	NA
	Blain 2002	107 Osteoporotic	68.9 ± 8.7	Total body	YSM, LM, FM, creatinine clearance, E2, leptin, DHEA, IGF-I, calcium intake	0.46	YSM, LM, leptin	0.272	**< 0.001**
				Femoral neck		0.376	YSM, LM, leptin	0.194	**< 0.05**
				Lumbar spine		0.305	YSM, LM, FM	NA	NA
	Cervellati 2016	127: normal(31), osteopenia(53), osteoporosis(43)	normal(55.3±0.7), osteopenia(56.7±0.6), osteoporosis(58.5±0.4)	Lumbar spine	leptin, age, waist circumference	NA	age, waist circumference	-0.08	NA
				Femoral neck				-0.01	
				Trochanter				-0.03	
				Total hip				0.07	
	Chanprasertyothin 2005	63	59.9 ± 7.9	Lumbar spine	Age, BMI, leptin	NA	Age, BMI	-0.07	NA
				Femoral neck				-0.05	
	Di Monaco 2003	62 Hip fractured	77.7 ± 8.1<br>62 - 93	Total hip, Unfractured femur	Age, weight, height, BMI, fracture type (cervical or trochanteric), time between fracture and laboratory analysis and DEXA assessment), Barthel index score, FM, LM, PTH, 25OHD, leptin	0.336	FM, leptin	-0.497	**< 0.01**
				Femoral neck, Unfractured femur		0.214		-0.38	**< 0.05**
	Jurimae 2008	88	68.9 ± 6.8<br>58 - 80	Total body	BMI, WHR, WTR, FM, FFM, ghrelin, adiponectin, leptin, IGF-I, insulin, HOMA	0.35	FM, FFM, leptin	0.001	**0.05**
				Femoral neck		0.22		0.002	**0.046**
	Ruhl 2002	1148	66 ± 0.6	Total hip	age, ethnicity, BMI, education, BMI change, calcium intake, TSH, postmenopausal oral estrogen use, leptin	NA	NA	0.01	0.69
	Värri 2016	290	73.6 ± 2.8 69.2-79.2	Total body	FM, age, smoking, E2, leptin	0.245	NA	0.029	0.739
					trunk fat mass, age, smoking, E2, leptin	0.27		-0.035	0.681
				Femoral neck	FM, age, smoking, E2, leptin	0.109		-0.097	0.304
					trunk fat mass, age, smoking, E2, leptin	0.132		-0.173	0.066
	Wu 2010	336	56.9 ± 6.5	Total Body	Age, YSM, BMI, FM, LM, smoking habits, calcium intake, physical activity, 25OHD, PTH, E2, T, insulin, adiponectin, leptin	0.198	YSM, LM, adiponectin, E2	NA	NA
				Lumbar spine		0.214	YSM, LM, adiponectin, E2, BMI		
				Total Hip		0.241	YSM, LM, adiponectin, E2		
				Total Forearm		0.244	YSM, LM, adiponectin		
	Yamauchi 2001	139	62.5 ± 7	Lumbar spine	leptin, %fat, age, YSM, height	NA	NA	0.006	0.087
				Femoral Neck				0.006	**0.027**
				1/3 radius				0.003	0.192
				Total Body				0.005	**0.028**
				Lumbar spine Z-score				0.062	0.069

				Femoral Neck Z-score				0.104	**0.015**
				1/3 radius Z-score				0.03	0.298
	Zhong 2005	336	NA	Lumbar spine	age, weight, BMI, leptin	0.001	NA	NA	NA
				Femoral Neck		0.026			
				Total hip		0.042			
				Left 1/3 radius+ulna		0.009			
	Zoico 2003	171	71.7 ± 2.4	Total Body	Age, leptin, FM, FFM	0.106	leptin	0.333	**< 0.001**
					Age, leptin, FM, FFM, BMI	0.132	BMI	NA	NA
	Zoico 2008	36	70.8 ± 2.6<br>66 - 77	Total body	BMI, FM, leptin, adiponectin, HOMA, DHEAS	0.387	FM, adiponectin	NA	NA
				Femoral neck		0.174	adiponectin		

25OHD, 25 hydroxyvitamin D; BMI, body mass index; DHEA, dehydroepiandrosterone sulfate; E2, estradiol; FFM, fat free mass; FM, fat mass; HOMA, homeostasis model assessment; LM, lean mass; NA, not applicable; PTH, parathormone; T, testosterone; WHR, waist-to-hip ratio; WTR, waist-to-thigh ratio; YSM, years since menopause.
